# A Green Processing Strategy for the Formation of Electrochromic Metal–Organic Assemblies

**DOI:** 10.1021/acs.langmuir.6c00947

**Published:** 2026-04-28

**Authors:** Anirban Chandra, Naveen Malik, Tanmoy Mandal, Yonatan Hamo, Linda J. W. Shimon, Tatyana Bendikov, Olga Brontvein, Graham de Ruiter, Michal Lahav, Milko E. van der Boom

**Affiliations:** † Department of Molecular Chemistry and Materials Science, 34976 Weizmann Institute of Science, Rehovot 7610001, Israel; ‡ Department of Chemical Research Support, 34976 Weizmann Institute of Science, Rehovot 7610001, Israel; § Schulich Faculty of Chemistry, 26747 Technion − Israel Institute of Technology, Technion City, Haifa 3200008, Israel

## Abstract

A key challenge in the development of electrochromic coatings is establishing fabrication methods that utilize sustainable materials and green solvents. This must be achieved while also optimizing multiple parameters, including color contrast, stability, switching time, and optical transmittance. In this study, we report water-soluble, redox-active iron-polypyridyl complexes as building blocks for electrochromic coatings and devices fabricated by layer-by-layer spin coating and automated spray coating. The solubility of these complexes in polar and nonpolar media was modulated by tuning the counterions (PF_6_^–^, Cl^–^, and SO_4_^2–^), without affecting the electrochromic performance of their assemblies. Furthermore, the use of two water-soluble palladium complexes, [PdCl_4_]^2–^ and *trans*-[Pd­(3-SO_3_H-py)_2_Cl_2_], allowed us to demonstrate that the resulting molecular assemblies can be formed using aqueous solutions. Overall, the molecular assemblies exhibited excellent optical and electrochromic properties with switching times between 0.9 and 1.8 s and up to 95% charge retention even after 2000 cycles. The practicality of our approach was demonstrated by fabricating laminated devices that retain the physicochemical properties of the original molecular assemblies.

## Graphical abstract

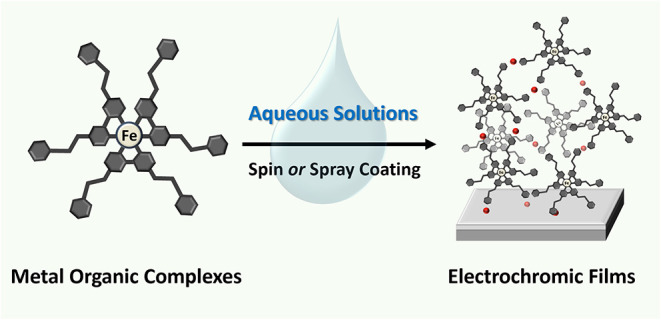

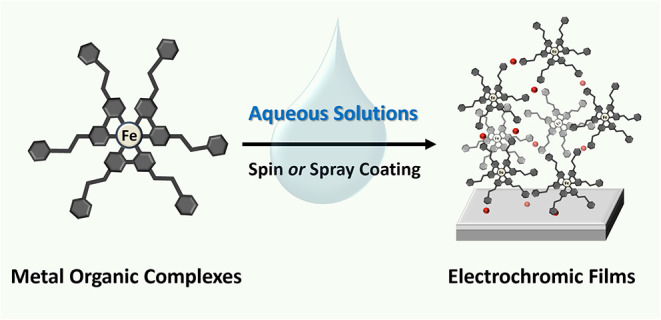

## Introduction

Over the past decades, a wide variety of electrochromic (EC) materials have been developed,^1–5^ including metal oxides,^6–8^ conductive polymers,^9,10^ liquid crystals,^1^ organic molecules,^2^ and metallo-supramolecular polymers.^3–8^ While most studies have focused on materials performance, one of the main bottlenecks in translating EC technologies from laboratory to large-scale fabrication lies in the processing stage. Current environmental regulations increasingly mandate the use of green solvents in production.^9–13^ Conventional fabrication techniques of EC coatings commonly employ organic, chlorinated, or aromatic solvents. Although these solvents enable homogeneous thin-film formation, they also present significant hazards due to their flammability, volatility, and toxicity. The adoption of green solvents, ideally water, offers a safer and more sustainable alternative.^14–18^ However, replacing organic solvents poses a major challenge, particularly for materials that are inherently insoluble in water or other green media. Adapting current protocols for sustainable and environmentally friendly (i.e., green) EC technologies requires the molecular redesign of electrochromic building blocks and the development of new coating methodologies that comply with current regulatory protocols.^19–23^ Reynolds and co-workers made a pioneering contribution by introducing environmentally benign solvent-processable coatings based on conducting organic polymers, a milestone in sustainable EC materials research.^9,10,24,25^ Unfortunately, the synthesis of water-soluble organic polymers remains nontrivial, as many such materials are intrinsically hydrophobic due to the presence of long alkyl side chains.^10,26,27^ Consequently, to achieve water solubility and maintain ionic conductivity in aqueous electrolytes, the polarity of these polymers must be tuned through structural modification.^24,28–31^

Our group has extensively explored coordination-based assemblies for the development of effective and versatile functional materials. These systems have demonstrated applications in heterogeneous catalysis,^32^ energy storage,^33^ Boolean logic,^34^ sensing,^35^ molecular transport,^36^ and EC devices.^4^ Over the years, various fabrication methods have been utilized, including dip coating, which yields exponential film growth,^37^ and more recently, automated spin^38^ and spray coating.^39^ However, adapting these methodologies to environmentally benign fabrication techniques remains challenging due to the limited aqueous solubility of the metal complexes and cross-linkers. Fortunately, the solubility of metal-polypyridyl complexes and polymers can be varied as a function of the counterions.^40,41^

In this study, we developed iron-polypyridyl complexes (**1** and **2**) with different counterions that exhibit enhanced solubility in eco-friendly solvents such as water, methanol or water–methanol mixtures (Scheme 1). By varying the counterion, we achieved high aqueous solubility for complexes bearing chloride (Cl^–^; **1**) and sulfate (SO_4_^2–^; **1**, **2**) anions, while those containing hexafluorophosphate (PF_6_^–^) remained insoluble in water. By using water-soluble palladium-based cross-linkers; *trans*-[Pd­(3-SO_3_H-py)_2_Cl_2_] (**3**) and [PdCl_4_]^2–^ (**4**), we were able to develop an aqueous fabrication process for the formation of EC coatings. By using layer-by-layer deposition of aqueous solutions of polypyridyl iron complexes (**1** or **2**) and palladium salts (**3** or **4**) up to 300 nm thick molecular assemblies could be formed that demonstrated excellent coloration efficiencies (258 cm^2^ C^–1^) combined with excellent stability (>2000 cycles). With an optical contrast of up to 49%, and response times of 0.9–1.8 s these EC coatings could be readily integrated into laminated devices that demonstrate the applicability of our approach.

**Scheme 1:**
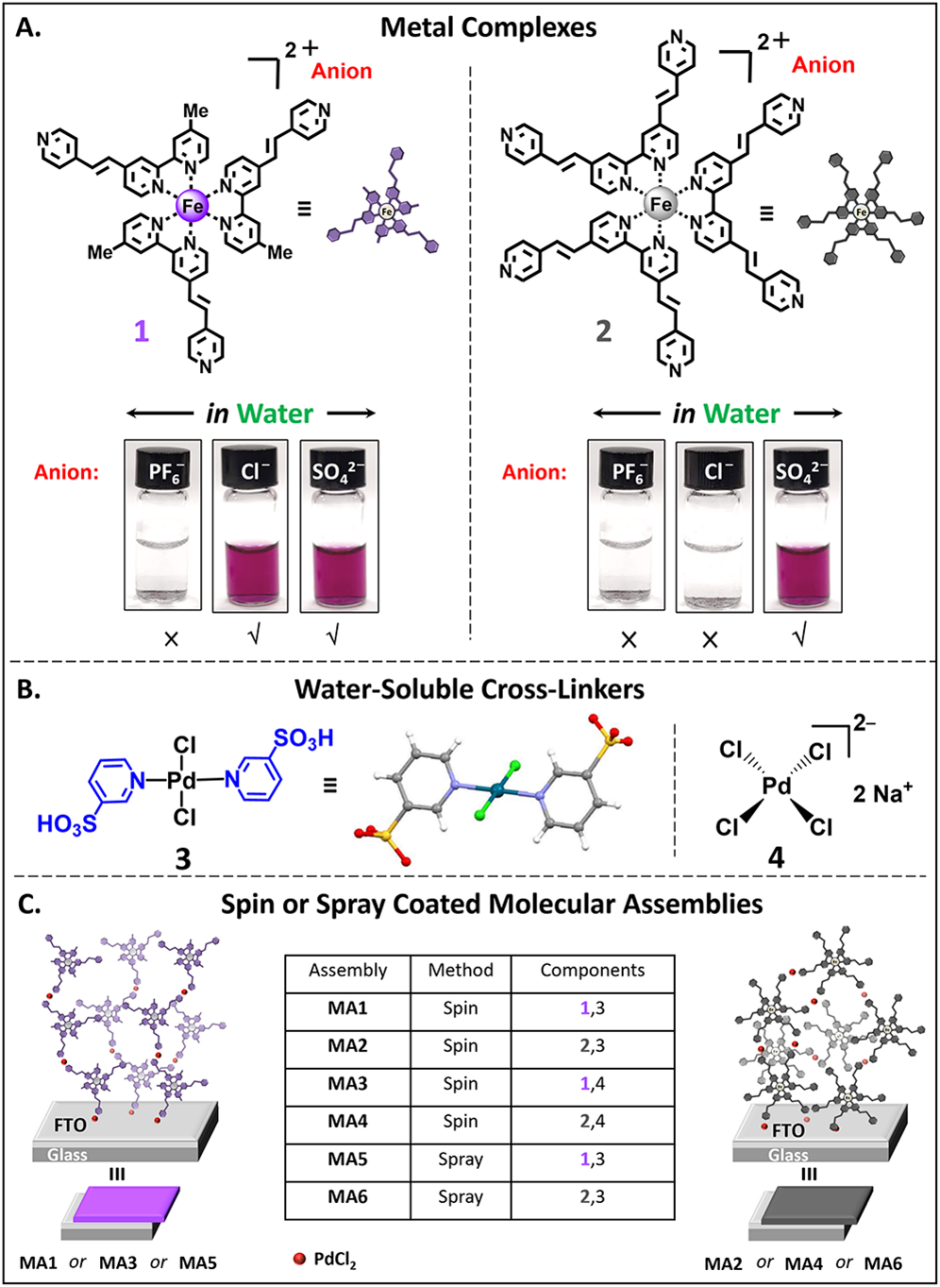
Molecular Structures and Assembly of Electrochromic Materials^a^

a (A) Molecular structures of the electrochromic complexes and photos showing their water solubility as a function of the counter anion. (B) Molecular structures of two divalent palladium cross-linkers (**3**,**4**), and the solid-state structure of complex **3**. (C) Molecular assemblies (MAs) based on pyridine coordination chemistry fabricated by alternating spin coating (**MA1-MA4**) or spray coating (**MA5-MA6**).

## Results and Discussion

The iron polypyridyl complexes (**1-Cl**, **1-SO**_**4**_ or **2-Cl**, **2-SO**_**4**_) were obtained in good yields (>80%) by reacting three equivalents of (*E*)-4-methyl-4′-(2-(pyridin-4-yl)­vinyl)-2,2′-bipyridine (**L1**) or 4,4′-bis­[(*E*)-2-(4-pyridyl)­vinyl]-2,2′-bipyridine (**L2**) with one equivalent of FeCl_2_·4H_2_O or FeSO_4_·7H_2_O. The complexes **1** and **2** exhibit spectroscopic (NMR and UV–vis) and electrochemical properties similar to our previously reported PF_6_^–^ analogs.^39^ The identity of the counterions in complexes **1** and **2** were confirmed by mass spectrometry and IR spectroscopy, as well as by their solubility behavior (Scheme 1A; see Supporting Information and Figures S1–S5 for details). Whereas **1-PF**_**6**_ and **2-PF**_**6**_ are insoluble in water, replacement by Cl^–^ or SO_4_^2–^ significantly alters their solubility. Complexes **1-Cl**, **1-SO**_**4**_, and **2-SO**_**4**_ are water-soluble, **2-Cl** only sparingly so. To render our entire fabrication process environmentally friendly, a new water-soluble palladium cross-linker *trans*-[Pd­(3-SO_3_H-py)_2_Cl_2_] (**3**) was developed as well, which was prepared in 80% yield by reacting two equivalents of pyridine-3-sulfonic acid with one equivalent of palladium­(II) chloride. Single-crystal X-ray diffraction (SCXRD) analysis shows that the pyridine-3-sulfonic acid ligands in **3** are coordinated to the *d* ^8^ palladium center in a mutually trans configuration (Scheme 1B; see Table S1 for more details). For comparison, we also evaluated the use of the commonly available water-soluble sodium salt of [PdCl_4_]^2–^ (**4**) as a cross-linker.

Our new EC molecular assemblies (**MA1-MA4**) were prepared from the polypyridyl iron complexes **1-SO**_**4**_ and **2-SO**_**4**_ and the two cross-linkers **3** and **4**. In brief, aqueous solutions of the individual components were sequentially spin-coated onto fluorine-doped tin oxide (FTO) glass substrates (2 cm × 2 cm). The substrates were first coated with the palladium cross-linkers **3** or **4**, which bind to surface hydroxyl groups on the FTO to form a dense anchoring layer. (see Supporting Information for experimental details). This layer-by-layer assembly procedure results in coordination networks by coordination of the palladium cations to the vinyl pyridyl moieties of the metal complexes.^4,39^ The resulting molecular assemblies (**MA1-MA4**) were characterized by UV–vis spectroscopy, focused ion beam–scanning electron microscopy (FIB-SEM), X-ray photoelectron spectroscopy (XPS), and electrochemical methods including spectroelectrochemistry (SEC).

The UV–vis spectra of **MA1**-**MA4** exhibits the expected metal-to-ligand charge-transfer (MLCT) bands (Figures 1 and S7). For **MA2** and **MA4**, two MLCT bands are observed at λ_max1_ = 455 nm and λ_max2_ = 596 nm, whereas **MA1** and **MA3** display a single MLCT band at λ_max_ = 579 nm. In addition, an intense π–π* transition appears at λ_max_ ≈ 330 nm for all MAs.^4,39^ Assuming that the absorption coefficients of the complexes **1** (ε = 2.2 × 10^4^ M^–1^ cm^–1^) and **2** (ε = 3.6 × 10^4^ M^–1^ cm^–1^) are similar to those in the thin films, the molecular surface densities were calculated ≈0.9 × 10^16^ molecules/cm^2^ (**MA1**), ≈1.0 × 10^16^ molecules/cm^2^ (**MA2**), ≈1.8 × 10^16^ molecules/cm^2^ (**MA3**), and ≈1.9 × 10^16^ molecules/cm^2^ (**MA4**); Table S2). For all MAs, the intensities of the absorption bands increase linearly with the number of deposition cycles, indicating the formation of a homogeneous structure along the *z*-direction (Figures 1A inset; Chart 1, 2 and S7A inset; Chart 1, 2). If redissolution or desorption of previously deposited layers were to occur during subsequent processing steps, such linear growth would not be expected.

**Figure 1:**
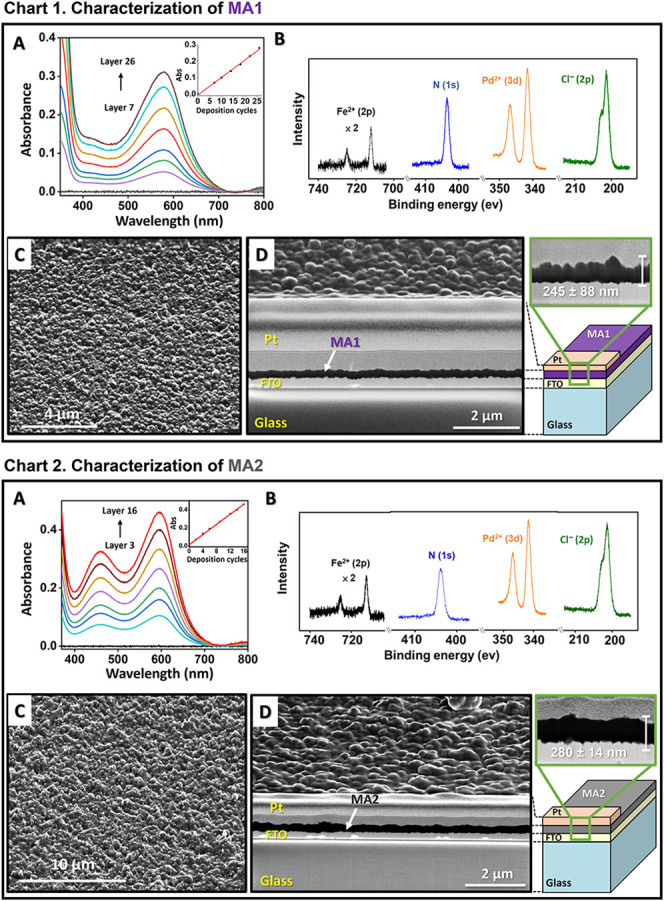
Characterization of [**MA1**|FTO/glass] (**Chart 1**) and [**MA2**|FTO/glass] (**Chart** 2). The **MA1** and **MA2** were prepared by alternating spin coating of aqueous solutions of *trans*-[Pd­(3-SO_3_H-py)_2_Cl_2_] followed by complex **1** or **2**, respectively. **Chart 1** and **Chart 2**: (A) Ex situ absorption spectra recorded during the formation of the **MA1** and **MA2**. A bare FTO substrate was used for the baseline (black). Inset: Absorbance intensity of the MLCT band (**MA1**: λ_max_ = 579 nm) or (**MA2**: λ_max_ = 596 nm) vs the number of deposition cycles. (B) X-ray photoelectron spectroscopy (XPS) spectra showing the Fe^2+^ 2p, N 1s, Pd^2+^ 3d, and Cl^–^ 2p regions. (C) Scanning electron microscopy (SEM) image of film surface. (D) Cross-section, obtained by milling with a 30 keV Ga^+^ focused ion beam (FIB). Pt coating was used to prevent ion beam damage, and the surface was first covered with a 3 nm thick layer of iridium.

SEM measurements illustrate the homogeneity and continuity of the grainy surfaces at the microscale. Information about the thickness and inner structure of the MAs was obtained by FIB-SEM measurements. Cross sections were obtained by milling the films with a FIB. Prior to this milling process, a platinum coating 0.6–0.8 μm was deposited to prevent damage caused by the ion-beam bombardment. Overall, the thickness of the MAs ranges from 245 to 295 nm, which is greater than the interfacial roughness of the coatings and the FTO (Figures 1C,D; Chart 1, 2 and S7C,D; Chart 1, 2). In all MAs, no apparent defects were observed, consistent with high-quality thin-film formation. XPS data confirmed the elemental composition. The presence of the divalent iron complex **1** or **2** is shown by two peaks of the 2p orbitals of Fe^2+^ at 708.5 eV (2p_3/2_) and 721.2 eV (2p_1/2_), while the two distinctive peaks of the 3d orbitals of Pd­(II) are observed at 337.9 eV (3d_5/2_) and 343.2 eV (3d_3/2_). The N1s signal is observed at 399.8 eV (Figures 1B; Chart 1, 2 and S7B; Chart 1, 2).^38,39^

The cyclic voltammograms (CVs) of **MA1**-**MA4** show reversible one-electron redox processes as expected for the Fe^2+/3+^ couple with half-wave potentials (*E*_1/2_) of 0.72 V for [**MA1**|FTO/glass], 0.67 V for [**MA2**|FTO/glass], 0.61 V for [**MA3**|FTO/glass], and 0.72 V for [**MA4**|FTO/glass] (Table S2). Based on the calculated current densities, the molecular densities were estimated at ≈0.7 × 10^16^ molecules/cm^2^ for [**MA1**|FTO/glass], ≈0.9 × 10^16^ molecules/cm^2^ [**MA2**|FTO/glass], ≈1.0 × 10^16^ molecules/cm^2^ [**MA3**|FTO/glass], and ≈1.2 × 10^16^ molecules/cm^2^ [**MA4**|FTO/glass] (Table S2), which is in good agreement with the values derived from the UV–vis data (vide supra). To further understand the electron transfer processes, we also varied the scan rate (ν) from 0.05 to 0.9 V/s (Figures S6A; Chart 1, 2 and S9A; Chart 1, 2). The anodic and cathodic peak currents (*I*_pa_ and *I*_pc_) show an exponential and linear correlation, when plotted against the scan rate and the square root of the scan rate (Figures S6B; Chart 1, 2 and S9B; Chart 1, 2). These electrochemical properties show that the electron-transfer processes are limited by diffusion of the electrolyte.^39,42–44^ During electrochemical cycling in LiClO_4_ electrolyte, counterion transport is required to maintain charge balance; thus, ion exchange between the initially incorporated counterions (SO_4_^2–^) and ClO_4_^–^ is likely. The calculated diffusion coefficient (*D*_f_) associated with the oxidation and reduction of the iron metal centers in [**MA1** and **MA3** |FTO/glass] varies between ≈1.5 × 10^–8^ cm^2^/s and ≈2.1 × 10^–8^ cm^2^/s, respectively (Table S2). By contrast, the *D*_f_ values for [**MA2** and **MA4** |FTO/glass] vary between ≈ 1.2 × 10^–9^ cm^2^/s and ≈2.4 × 10^–9^ cm^2^/s (Table S2). The differences might imply that the molecular packing of the structurally differentiated complexes (**1**, **2**) with different Pd­(II) linkers affects the diffusion of anions in the aqueous LiClO_4_ solution. Differences in anion size and coordination properties may influence the extent and kinetics of this process.^4,39^ More densely packed films were obtained when the [PdCl_4_]^2–^ (**4**) was used as cross-linker (Table S2). The number of vinyl pyridine groups (3 vs 6) in the complex is also likely to play a role.

The electrochemical and SEC properties of **MA1**-**MA4** assembled on FTO/glass were assessed using a three-electrode cell configuration consisting of the coated FTO as the working electrode and the Pt and Ag/Ag^+^ wires as the counter and quasi-reference electrodes, respectively (Figures 2 and S8). LiClO_4_ (0.1 M) in water was used as an electrolyte. The color changes of the MAs, upon oxidation (1.2 V) and reduction (0.2 V) of the iron complexes, are clearly visible to the naked eye, as shown in the photographs (Figures 2A; Chart 1, 2 and S8A; Chart 1, 2). **MA1** and **MA3** are purple in color, whereas **MA2** and **MA4** are gray in their reduced states, and transparent when oxidized. The corresponding Δ*T*% values were calculated at their characteristic MLCT absorption bands (λ_max_), which are in the range of 39–47% for **MA1-MA4** (Figures 2B,C; Chart 1, 2 and S8B,C; Chart 1, 2). These values are dependent on the switching times, after ≈2 s the maximum Δ*T*% is achieved. SEC measurements show ≥95% retention of the initial Δ*T*% for ≈ 2000 cycles for **MA1**, **MA2**, and **MA4**. However, for **MA3** ≥ 95% retention of the initial Δ*T*% is observed for only 100 cycles. These two cross-linkers with different geometries provide significant effects on the electrochemical performance for complex **1**. Finally, to demonstrate the scope of our methodology, we show that spray-coated methanolic solutions of complexes **1** and **2** yield molecular assemblies with comparable electrochromic performance to spin-coated **MA1** and **MA2**, exhibiting similar Δ*T*% values of 49% (**MA5**) and 42% (**MA6**), as well as comparable stability (Figure S10).

**Figure 2:**
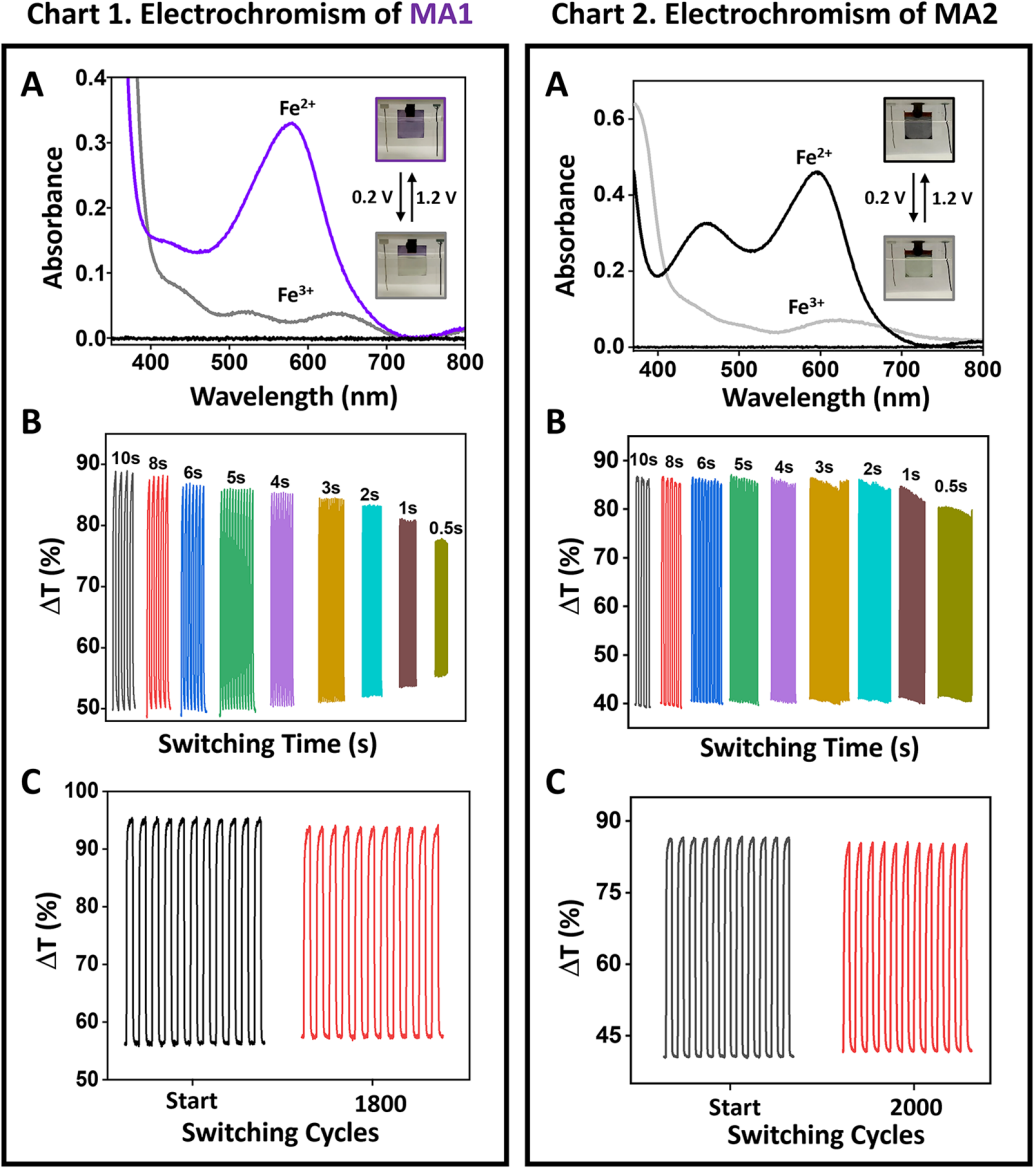
Electrochromic properties of [**MA1**|FTO/glass] and [**MA2**|FTO/glass] in 0.1 M LiClO_4_/H_2_O electrolyte solution using Pt wire and Ag/Ag^+^ wire, as counter and quasi-reference electrodes, respectively. The **MA1** and **MA2** were prepared by alternating spin coating of aqueous solutions of *trans*-[Pd­(3-SO_3_H-py)_2_Cl_2_] and complex **1** and **2**, respectively. **Chart 1:** [**MA1**|FTO/glass] and **Chart 2**: [**MA2**|FTO/glass]: (A) Absorption spectra showing the reduced (0.2 V) and oxidized (1.2 V) states. FTO/glass was used for the baseline (black). Inset: Photographs of the colored (Fe^*2*+^, 0.2 V) and bleached (Fe^*3*+^, 1.2 V) states. (B) Spectroelectrochemical (SEC) measurements at different switching times. (C) SEC stability measurements. For (B) and (C) Double potential steps: 0.2 to 1.2 V, λ_max_ = 579 nm (**Chart 1**) and λ_max_ = 596 nm (**Chart 2**).

The electrochromic properties of the spin-coated **MA1** and **MA2** were also evaluated in laminated device architectures with optical windows of 1.7 cm × 1.3 cm (Figure 3). This setup consisted of (i) [**MA**|FTO/glass] as the working electrode (bottom), (ii) FTO/glass as the counter and reference electrodes (top), (iii) a poly­(methyl methacrylate)-(PMMA)-based gel electrolyte, and (iv) double-sided tape (3 M 9088) as an insulating spacer. **MA1** and **MA2**-based devices exhibit large changes in color intensity between the colored (reduced) and bleached (oxidized) states with Δ*T*_max_ ≈ 36% at λ_max_ = 596 nm (gray) or λ_max_ = 579 nm (purple). Furthermore, these low-voltage operable (from −2.0 to +3.0 V) electrochromic devices (ECDs) were reversibly addressed up to ≈1000 redox cycles (≥95% stability) with a pulse width of 8 s.

**Figure 3:**
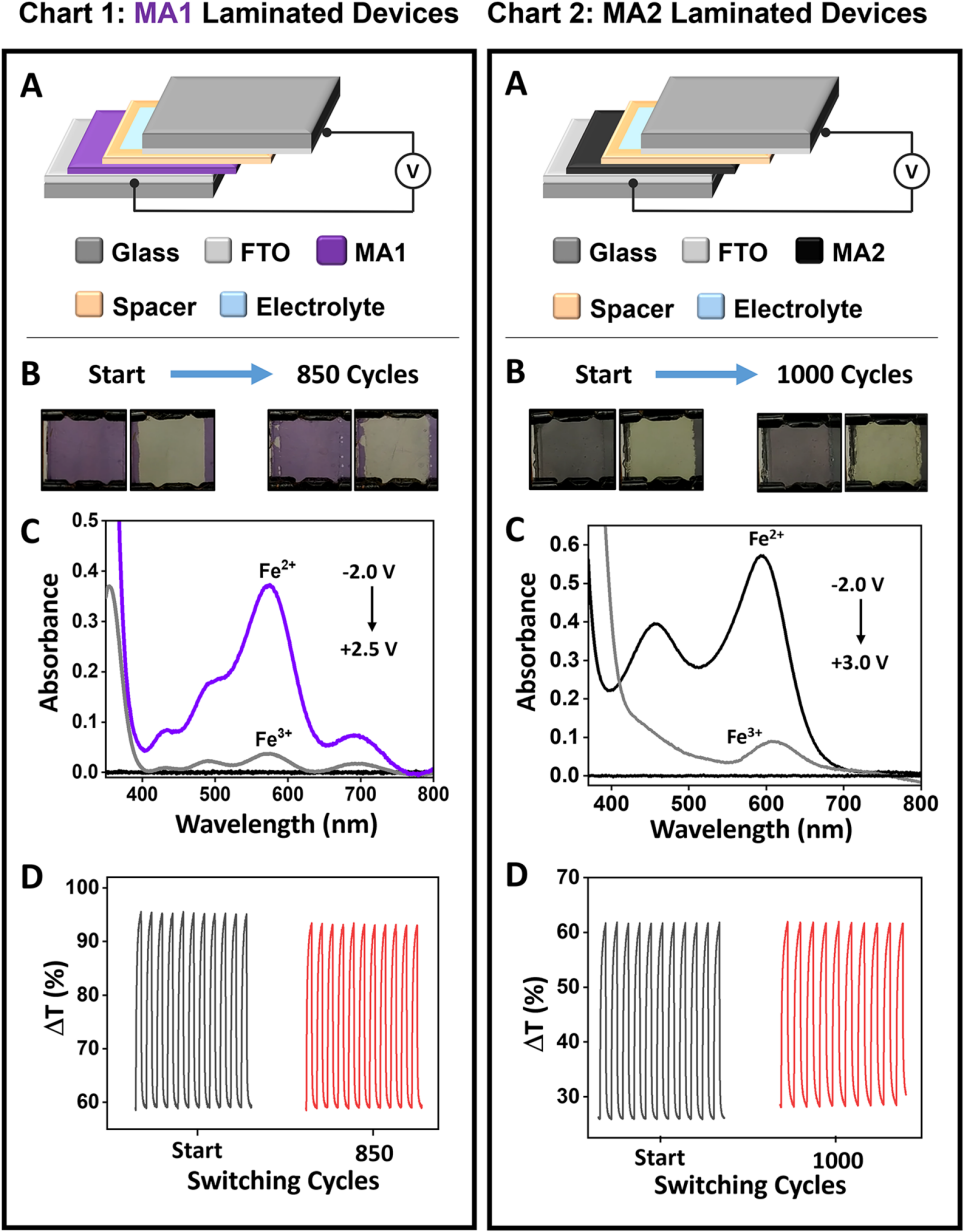
Spectroelectrochemical (SEC) performance of laminated electrochromic devices (ECDs) based on [**MA1**|FTO/glass] and [**MA2**|FTO/glass] as the working electrode and bare FTO as the counter electrode (CE) using electrolyte gel (90:7:3 wt % ACN/PMMA/lithium perchlorate salt). **Chart 1:** [**MA1**|FTO/glass] and **Chart 2**: [**MA2**|FTO/glass]: (A) Schematic representation of the device structures. (B) **Chart 1**: Photographs of the colored (Fe^2+^, −2.0 V) and bleached (Fe^3+^, + 2.5 V) states of **MA1**-ECD, initially and after 850 redox cycles, and **Chart 2**: photographs of the colored (Fe^2+^, −2.0 V) and bleached (Fe^3+^, + 3.0 V) states of **MA2**-ECD, initially and after 1000 redox cycles. (C) Absorption spectra showing the reduced and oxidized states. FTO/glass was used for the baseline (black). (D) SEC stability measurements at λ_max_ = 579 nm (**Chart 1**) using double potential steps: +2.5 to −2.0 V, and at λ_max_ = 596 nm (**Chart 2**) using double potential steps: −2.0 to +3.0 V.

## Conclusions

This study demonstrates that replacing the anions of iron polypyridyl complexes provides an effective strategy for fabricating electrochromic coatings in environmentally benign solvents. Although methanol is used in specific steps, the process remains predominantly aqueous and substantially reduces the use of organic solvents. By exploiting the anion-solubility relationship, water-based processing can be achieved without modifying the molecular structures of the complexes. The resulting coatings exhibit electrochromic performance comparable to those prepared with organic solvents, also using pyridine coordination chemistry.^4,39^ The structure and properties of the assemblies (**MA1**-**MA4**) can be rationalized based on two primary parameters: (i) the nature of the electrochromic complex (**1** vs **2**) and (ii) the type of Pd-based cross-linker (**3** vs **4**), which together influence film density, connectivity, and ion transport. The electrochromic complex primarily determines the optical properties (color and λ_max_), while the cross-linker governs film formation and transport characteristics, as reflected in differences in switching times and diffusion coefficients (Table S2). Variations in the number of electrochromic molecules per unit area (derived from UV–vis and CV measurements) indicate differences in film density, which correlate with ion transport and, consequently, electrochemical performance. Water-soluble palladium cross-linkers, [PdCl_4_]^2–^ and *trans*-[Pd­(3-SO_3_H-py)_2_Cl_2_], enabled the formation of robust films showing reversible color-to-colorless transitions with high optical contrast and efficiency at low operating voltages. The latter cross-linker produced better electrochromic properties, emphasizing the importance of cross-linker structure and reactivity. Notably, the laminated ECDs operated reliably for 850–1000 cycles without dedicated ion-storage layers,^33,39^ simplifying fabrication and avoiding the optical losses often associated with such layers. Overall, this approach offers an efficient pathway for developing sustainably processed electrochromic materials by using well-established coating methods. In addition, it establishes palladium–pyridine coordination chemistry as a viable platform for electrochromic materials under aqueous conditions and highlights the broader applicability of counterion engineering for enabling aqueous on-surface coordination assembly.^4,34,45–50^

## Experimental Section

### Materials and Methods

Solvents (AR grade) were purchased from Bio-Lab (Jerusalem, Israel), Frutarom (Haifa, Israel), or Mallinckrodt Baker (Phillipsburg, NJ). Ferrous sulfate heptahydrate (FeSO_4_·7H_2_O), ferrous chloride tetrahydrate (FeCl_2_·4H_2_O), palladium dichloride (PdCl_2_), sodium tetrachloropalladate (Na_2_PdCl_4_), lithium perchlorate (LiClO_4_), ammonium chloride (NH_4_Cl), ammonium sulfate (NH_4_)_2_SO_4_, and poly­(methyl methacrylate) (PMMA) were purchased from Merck. The ligands **L1**, **L2**, and iron complexes **1-PF**_**6**_, **2-PF**_**6**_ were synthesized according to literature procedures.^39^ Ultrapure (Type 1) water was obtained from Millipore Synergy water purification system. Fluorine-doped tin oxide (FTO)-coated glass substrates (2 cm × 2 cm, Rs = 10 Ω/□) were purchased from Xinyan Technology Ltd. (Hong Kong, China). FTO-coated glass substrates were cleaned by sonication in ethanol for 10 min, dried under a stream of N_2_, and subsequently cleaned for 20 min in a UVOCS cleaning system (Montgomery, PA). The substrates were then rinsed with tetrahydrofuran (THF), dried under a stream of N_2_, and oven-dried at 130 °C for 2 h prior to use. Molecular assemblies (MAs) on FTO substrates were formed either by (i) spin coating, using a Laurell WS-65MZ-8NPPB spin-coater or by (ii) spray coating using an automatic Ultrasonic Spraying System (Sono-Tek) equipped with two ultrasonic nozzles (having 2–6 mm diameter spray areas, operating at 120 kHz), mounted onto an X-Y-Z movable scanner. UV–vis spectra (absorbance and transmittance) were recorded on a Cary 100 spectrophotometer using the Cary WinUV-Scan and WinUV-Kinetics application program. Unfunctionalized FTO substrates were used to correct for the background absorption. IR spectra were recorded using a Nicolet iS50 FTIR Spectrometer in the iS50 ATR mode with OMNIC software.

### Synthesis of Complexes **1-Cl** and **1-SO_4_**

A solution of (*E*)-4-methyl-4′-(2-(pyridin-4-yl)­vinyl) 2,2′-bipyridine **L1** (2.73 g, 10 mmol, 3 equiv) MeOH (10 mL) was added to a solution of FeCl_2_.4H_2_O (0.65 g, 3.3 mmol, 1 equiv) or FeSO_4_·7H_2_O (0.92 g, 3.3 mmol, 1 equiv) in MeOH (10 mL). The solution was then stirred at 50 °C. After 30 min, the solvent was removed under reduced pressure, and the resulting crude solid was suspended in diethyl ether (200 mL). The suspension was subsequently filtered, and the collected solid was washed with additional diethyl ether (200 mL). Finally, the solid was collected to yield the title compound as a purple color solid. Yield **1-Cl**: 80%; **1-SO**_**4**_: 82%.

#### Complex **1-Cl**

^1^H NMR: (500 MHz, CD_3_OD) δ 9.02 (bs, 3H), 8.83 (s, 3H), 8.60 (s, 6H), 7.86 (d, *J* = 16.2 Hz, 3H), 7.71 (d, *J* = 16.4 Hz, 12H), 7.62–7.48 (m, 3H), 7.37 (m, 6H), 2.66 (s, 9H). ^13^C­{^1^H} NMR (126 MHz, CD_3_OD) δ 161.3, 160.0, 155.2, 154.0, 153.0, 150.7, 147.9, 145.8, 134.8, 130.5, 129.8, 126.4, 125.8, 123.2, 122.2, 21.2. HRMS (ESI^+^, *m*/*z*): calcd. for [C_54_H_45_N_9_Fe]^2+^ 437.6568; found 437.6537.

#### Complex **1-SO_4_**

^1^H NMR (500 MHz, CD_3_OD) δ 9.02 (s, 2H), 8.84 (s, 2H), 8.68–8.47 (m, 8H), 7.91–7.46 (m, 18H), 7.36 (d, *J* = 28.4 Hz, 6H), 2.58 (s, 9H). ^13^C­{^1^H} NMR (126 MHz, CD_3_OD) δ 161.4, 160.1, 155.1, 153.9, 153.1, 150.8, 147.9, 145.7, 134.8, 130.6, 126.5, 125.7, 123.3, 122.4, 21.3. HRMS (ESI^+^, *m*/*z*): calcd. for [C_54_H_45_N_9_Fe]^2+^ 437.6568; found 437.6520.

### Synthesis of Complexes **2-Cl** and **2-SO_4_**

A suspension of 4,4′-bis­[(*E*)-2-(4-pyridyl)­vinyl]-2,2′-bipyridine **L2** (3.62 g, 10 mmol, 3 equiv) in MeOH (50 mL) was added to a solution of the metal salt of FeCl_2_·4H_2_O (0.65 g, 3.3 mmol, 1 equiv) or FeSO_4_·7H_2_O (0.92 g, 3.3 mmol, 1 equiv) in MeOH (10 mL). The solution was then stirred at 50 °C. After 30 min, the solvent was removed under reduced pressure, and the resulting crude solid was suspended in diethyl ether (200 mL). The suspension was subsequently filtered, and the collected solid was washed with additional diethyl ether (200 mL). Finally, the solid was collected to yield the title compound as a grayish-black color solid. Yield **2-Cl**: 85%; **2-SO**_**4**_: 84%.

#### Complex **2-Cl**

^1^H NMR (500 MHz, CD_3_OD) δ 9.19 (bs, 3H), 8.73 (d, *J* = 4.9 Hz, 3H), 8.66–8.57 (m, 12H), 7.90 (d, *J* = 16.4 Hz, 6H), 7.75–7.62 (m, 30H). ^13^C­{^1^H} NMR (126 MHz, CD_3_OD): δ 161.0, 157.6, 155.2, 150.6, 146.8, 135.2, 132.5, 130.4, 123.1, 120.3. HRMS (ESI^+^, *m*/*z*): calcd. for [C_72_H_54_N_12_Fe]^2+^ 571.1967; found 571.1946.

#### Complex **2-SO_4_**

^1^H NMR (500 MHz, CD_3_OD) δ 9.24 (bs, 3H), 8.73 (d, *J* = 4.9 Hz, 3H), 8.59 (dd, *J* = 9.8, 6.6 Hz, 12H), 7.90 (d, *J* = 11.1 Hz, 6H), 7.80–7.69 (m, 21H), 7.67–7.59 (m, 9H). ^13^C­{^1^H} NMR (126 MHz, CD_3_OD): δ 161.0, 157.6, 155.0, 150.6, 146.4, 135.1, 132.5, 130.4, 123.3, 120.3. HRMS (ESI^+^, *m*/*z*): calcd. for [C_72_H_54_N_12_Fe]^2+^ 571.1967; found 571.1946.

### Synthesis of Complex **3**

To an aqueous solution (10 mL) of pyridine-3-sulfonic acid (1.60 g, 10 mmol, 2 equiv), an aqueous solution (5 mL) of palladium­(II) chloride (0.88 g, 5.0 mmol, 1 equiv) was added. The resulting reaction mixture was heated at 80 °C for 2 h and subsequently filtered to remove any insoluble particulates. Hereafter, the solvent was removed under reduced pressure to obtain the title compound as a brown solid. Yield: 80%. Single crystals suitable for Single X-ray diffraction (SC-XRD) could be obtained by slow evaporation of H_2_O from a concentrated aqueous solution of **3**. ^1^H NMR (300 MHz, D_2_O) δ 9.13 (s, 2H), 8.88 (d, *J* = 5.6 Hz, 2H), 8.31 (d, *J* = 8.1 Hz, 2H), 7.65 (dd, *J* = 8.0, 5.8 Hz, 2H). ^13^C­{^1^H} NMR (126 MHz, D_2_O) δ 154.6, 149.7, 141.2, 137.1, 126.2. MS (ESI^+^), *m*/*z*: calcd. for [C_10_H_10_ClN_2_O_6_S_2_Pd]^+^ 460.18; found 460.87.

#### Formation of Molecular Assemblies MA1 and MA2 by Spin Coating

Molecular assemblies were prepared by alternating spin coating of aqueous solutions of **3** (4.0 mM) and solutions of either **1-SO**_**4**_ (0.6 mM) or **2-SO**_**4**_ (0.6 mM). For **2-SO**_**4**_, a H_2_O/MeOH mixture (95:5 v/v) was used. Assembly formation was initiated by drop casting a solution of **3** (0.6–0.7 mL, 4.0 mM) onto the substrate, followed by spinning at 500 rpm for 10 s and then at 1000 rpm for 60 s. After a stationary period of 80 s, a solution of **1-SO**_4_ (0.6–0.7 mL, 0.6 mM) for **MA1** or **2-SO**_**4**_ (0.6–0.7 mL, 0.6 mM) for **MA2** was drop cast onto the substrate, which was then spun using the same protocol. Next, the substrates were immersed in methanol (25 mL) for 30 s and dried under a gentle stream of air. A single deposition cycle consists of one layer of **3** followed by one layer of **1-SO**_**4**_ or **2-SO**_**4**_. For **MA1**, the number of deposition cycles was repeated 26 times, while for **MA2,** it was repeated 16 times in order to obtain the completed molecular assemblies.

#### Formation of Molecular Assemblies MA3 and MA4 by Spin Coating

The assemblies were obtained by alternatingly spin-coating aqueous solutions of Na_2_PdCl_4_ (**4**) followed by aqueous solutions of complex **1-SO**_**4**_ or complex **2-SO**_**4**_. Note that for **2-SO**_**4**_ a mixture of H_2_O:MeOH (95%:5% v/v) was used. Formation of the molecular assemblies was initiated by drop casting a solution of **4** (0.6–0.7 mL; 4.0 mM) onto the substrate, whereafter the substrate was spun at 500 rpm for 10 s, followed by 1000 rpm for 60 s. Hereafter, a solution (0.6–0.7 mL; 0.6 mM) of the **1-SO**_**4**_ (**MA3**) or **2-SO**_**4**_ (**MA4**) was drop-casted after 80 s onto the substrates, which were again spun according to the above-mentioned protocol. Next, the substrates were immersed in methanol (0.6–0.7 mL) for 30 s and dried under a gentle stream of air. The deposition of **4** followed by the deposition of **1-SO**_4_ or **2-SO**_**4**_ is referred to as a single deposition cycle. For the formation of **MA3**, the deposition cycle was repeated 26 times, whereas for the formation of **MA4**, the deposition cycle was repeated 18 times, respectively.

#### Formation of Molecular Assemblies MA5 and MA6 by Spray Coating

The assemblies were obtained by automated and alternatingly ultrasonic spray coating of a methanol solution of **3** (1.0 mM) and of complex **1-SO**_**4**_ or **2-SO**_**4**_ in methanol (0.2 mM), respectively. The coatings were formed on FTO/glass (2 cm × 2 cm) at an atomization pressure of 1.03 or 1.30 kPa. The nozzle-to-substrate distance was 5.5 cm, and the nozzle was moved in a preprogrammed pattern along the *X* and *Y* directions at a speed of 5 mm/s and with a flow rate of 0.6 mL/min at room temperature (∼23 °C). The methanol solution of **3** (1.0 mM) was sprayed onto the substrate (3 passes), which was followed by spraying (3 passes) the solutions complex **1-SO**_**4**_ or **2-SO**_**4**_ in methanol (0.2 mM). This deposition sequence was repeated six times to generate **MA5** and **MA6**. The substrates after formation of the **MAs** were removed from the spray coater and immersed in methanol for 30 s and dried under a gentle stream of air.

#### Fabrication of Electrochromic Devices

The FTO/glass substrates coated with **MA1**-**MA2** served as the working electrode, and a bare substrate (FTO/glass) served as both the reference and counter electrodes. A frame of 210-μm-thick double-sided tape (3 M 9088) was attached to the working electrode (2 cm × 2 cm) leaving an exposed edge (1–2 mm) for silver paste or copper tape contacts. Contacts were also connected to an edge (1–2 mm) of the counter electrode. The electrodes were placed with the two conducting faces facing each other. The electrolyte gel (90:7:3 wt % ACN/PMMA/lithium perchlorate salt) was injected using a syringe between the two electrodes.

## Supplementary Material





## Data Availability

All data supporting this study are provided as part of the article and its Supporting Information.

## Notes

Additional characterization data, Fourier-transform infrared spectroscopy, UV-vis spectra, and electrochemical data (PDF)
Single-crystal X-ray diffraction data of complex **3** (CIF)
