# Supplementary material for: A Green Processing Strategy for the Formation of Electrochromic Metal–Organic Assemblies
Source: Langmuir. 2026 Apr 28;42(18):12928–36. doi: 10.1021/acs.langmuir.6c00947 (PMC13178065; doi:10.1021/acs.langmuir.6c00947)
Supplement: Supplementary file 1 [file la6c00947_si_001.pdf]

Supporting Information

A green processing strategy for the formation of electrochromic metal-organic assemblies

Anirban Chandra[a,#], Naveen Malik[a,#], Tanmoy Mandal[a], Yonatan Hamo[a], Linda J. W. Shimon[b], Tatyana Bendikov[b], Olga Brontvein[b], Graham de Ruiter[c], Michal Lahav[a,*] and Milko E. van der Boom[a,*]

[a] Department of Molecular Chemistry and Materials Science, Weizmann Institute of Science, 7610001 Rehovot, Israel

[b] Department of Chemical Research Support, Weizmann Institute of Science, 7610001 Rehovot, Israel

[c] Schulich Faculty of Chemistry, Technion – Israel Institute of Technology, Technion City, Haifa 3200008, Israel

[#] Both authors contributed equally

[*] Corresponding authors.
Email: michal.lahav@weizmann.ac.il, milko.vanderboom@weizmann.ac.il

Table of Contents

Materials and Methods S2
X-ray Photoelectron Spectroscopy (XPS) S2
Scanning Electron Microscopy (SEM) and Focused Ion Beam (FIB) S3
Electrochemical Characterization S3
Synthesis of complexes **1-Cl**, **1-SO$_4$**, **2-Cl**, **2-SO$_4$** and **3** S4-S6
Single-crystal X-ray diffraction of complex **3** S6
Table S1. Single-crystal X-ray diffraction data of complex **3** S7
Formation of Molecular Assemblies **MA1-MA6** S8-S9
Fabrication of Electrochromic Devices. S9
Figure S1-S4. UV-Vis spectra and CVs of **1-Cl**, **1-SO$_4$**, **2-Cl** and **2-SO$_4$** S10-S11
Figure S5. Fourier-transform infrared spectroscopy (FTIR) spectra S12
Figure S6-S10. Characterization data of **MA1-MA6** S13-S17
Table S2. Comparison of various parameters for **MA1-MA4** S18
References S19

Experimental Section

Materials and Methods

Solvents (AR grade) were purchased from Bio-Lab (Jerusalem), Frutarom (Haifa, Israel), or Mallinckrodt Baker (Phillipsburg, NJ). Ferrous sulfate heptahydrate ($FeSO_4 \cdot 7H_2O$), ferrous chloride tetrahydrate ($FeCl_2 \cdot 4H_2O$), palladium dichloride ($PdCl_2$), sodium tetrachloropalladate (Na_2PdCl_4), lithium perchlorate ($LiClO_4$), ammonium chloride (NH_4Cl), ammonium sulfate ($(NH_4)_2SO_4$, and poly(methyl methacrylate) (PMMA) were purchased from Merck. The ligands **L1**, **L2** and iron complexes **1-PF$_6$**, **2-PF$_6$** were synthesized according to literature procedures.[1-2] Ultra-pure (Type 1) water was obtained from Millipore Synergy water purification system. Fluorine-doped tin oxide (FTO)-coated glass substrates (2 cm × 2 cm, Rs = 10 Ω/□) were purchased from Xinyan Technology Ltd. (Hong Kong, China). FTO-coated glass substrates were cleaned by sonication in ethanol for 10 min., dried under a stream of N_2, and subsequently cleaned for 20 min in a UVOCS cleaning system (Montgomery, PA). The substrates were then rinsed with tetrahydrofuran (THF), dried under a stream of N_2, and oven-dried at 130 °C for 2 h prior to use. Molecular assemblies (MAs) on FTO substrates were formed either by (i) spin coating, using a Laurell WS-65MZ-8NPPB spin-coater or by (ii) spray coating using an automatic Ultrasonic Spraying System (Sono-Tek) equipped with two ultrasonic nozzles (having 2–6 mm diameter spray areas, operating at 120 kHz), mounted onto an X–Y–Z movable scanner. UV-Vis spectra (absorbance and transmittance) were recorded on a Cary 100 spectrophotometer using the Cary WinUV−Scan and WinUV−Kinetics application program. Unfunctionalized FTO substrates were used to correct for the background absorption. IR spectra were recorded using a Nicolet™ iS50 FTIR Spectrometer in the iS50 ATR mode with OMNIC software.

X-ray Photoelectron Spectroscopy (XPS) XPS measurements were carried out on FTO/glass substrates (1.0 cm × 1.0 cm) with a Kratos AXIS ULTRA system, using a monochromatic Al Kα X-ray source (hυ = 1486.6 eV) at 75 W and detection pass energies ranging between 20 and 80 eV. Curve-fitting analysis was based on Shirley or linear background subtraction and applying Gaussian–Lorentzian line shapes.

Scanning Electron Microscopy (SEM) and Focused Ion Beam (FIB) SEM images were recorded using a Helios 600 FIB/SEM dual-beam microscope (FEI), operating at 5 keV. The images were taken at the surface of the samples and at cross sections that were milled with 30 keV Ga^{+}-FIB. The molecular assemblies deposited on FTO/glass were first coated with a 3-nm-thick layer of iridium (to enhance the conductivity of the surface), followed by coating a 150–200 nm thick layer of platinum using electron-beam-assisted deposition. This process was followed by the anion-beam-assisted deposition of a 500–600 nm thick layer of platinum. The platinum coating protects the molecular assemblies from ion-beam damage.

Electrochemical Characterization Electrochemical experiments were carried out using a CHI760E electrochemical workstation. The following configuration of the electrochemical cell was used. Working electrode: molecular assemblies deposited on FTO/glass (2 cm × 2 cm), reference electrode: Ag/Ag^{+}, and the counter electrode: Pt wire. Lithium perchlorate ($LiClO_4$, 0.1 M) in H_2O was used as the supporting electrolyte. The same electrodes were used for the electrochemical characterization of complexes **1** and **2** in solution

Synthesis of complexes 1-Cl and 1-SO$_4$

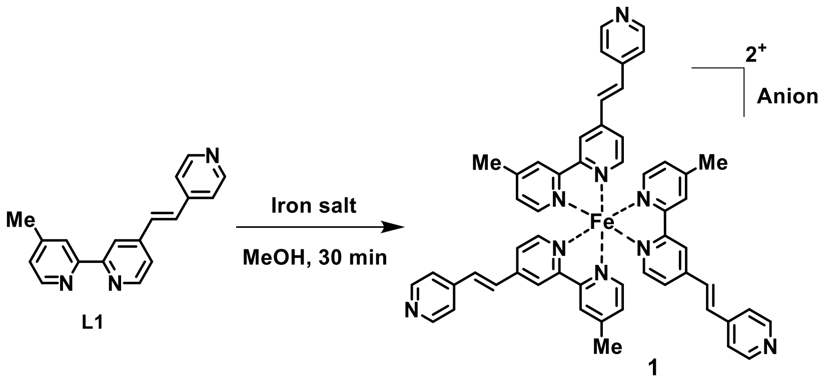

A solution of (*E*)-4-methyl-4'-(2-(pyridin-4-yl)vinyl)-2,2'-bipyridine **L1** (2,73 g, 10 mmol, 3 equiv.) MeOH (10 mL) was added to a solution of $FeCl_2{\cdot}4H_2O$ (0.65 g, 3.3 mmol, 1 equiv.) or $FeSO_4{\cdot}7H_2O$ (0.92 g, 3.3 mmol, 1 equiv.) in MeOH (10 mL). The solution was then stirred at 50 °C. After 30 min., the solvent was removed under reduced pressure, and the resulting crude solid was suspended in diethyl ether (200 mL). The suspension was subsequently filtered, and the collected solid was washed with additional diethyl ether (200 mL). Finally, the solid was collected to yield the title compound as a purple color solid. Yield **1-Cl**: 80%; **1-SO$_4$**: 82%.

Complex 1-Cl: ^{1}H NMR: (500 MHz, CD_3OD) δ 9.02 (bs, 3H), 8.83 (s, 3H), 8.60 (s, 6H), 7.86 (d, J = 16.2 Hz, 3H), 7.71 (d, J = 16.4 Hz, 12H), 7.62 – 7.48 (m, 3H), 7.37 (m, 6H), 2.66 (s, 9H). ^{13}C{^{1}H} NMR (126 MHz, CD_3OD) δ 161.3, 160.0, 155.2, 154.0, 153.0, 150.7, 147.9, 145.8, 134.8, 130.5, 129.8, 126.4, 125.8, 123.2, 122.2, 21.2. HRMS (ESI$^+$, *m/z*): calcd. for $[C_{54}H_{45}N_9Fe]^{2+}$ 437.6568; found 437.6537.

Complex 1-SO$_4$: ^{1}H NMR (500 MHz, CD_3OD) δ 9.02 (s, 2H), 8.84 (s, 2H), 8.68 – 8.47 (m, 8H), 7.91 – 7.46 (m, 18H), 7.36 (d, J = 28.4 Hz, 6H), 2.58 (s, 9H). ^{13}C NMR (126 MHz, CD_3OD) δ 161.4, 160.1, 155.1, 153.9, 153.1, 150.8, 147.9, 145.7, 134.8, 130.6, 126.5, 125.7, 123.3, 122.4, 21.3. HRMS (ESI$^+$, *m/z*): calcd. for $[C_{54}H_{45}N_9Fe]^{2+}$ 437.6568; found 437.6520.

Synthesis of complexes 2-Cl and 2-SO_4

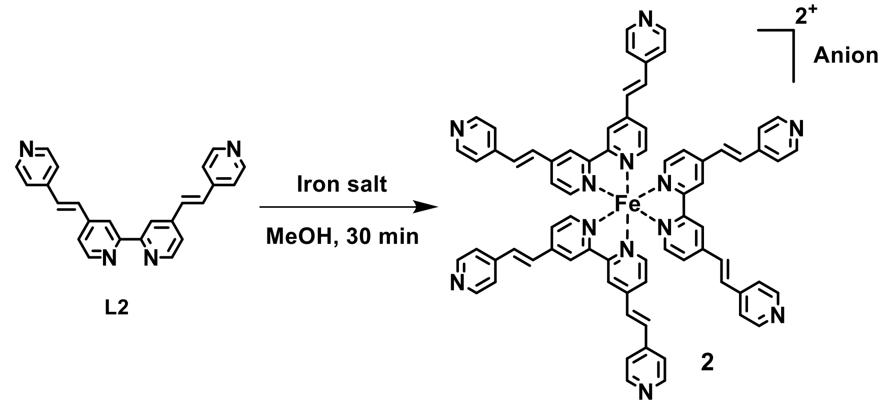

A suspension of 4,4'-bis[(*E*)-2-(4-pyridyl)vinyl]-2,2'-bipyridine **L2** (3.62 g, 10 mmol, 3 equiv.) in MeOH (50 mL) was added to a solution of the metal salt of $FeCl_2{\cdot}4H_2O$ (0.65 g, 3.3 mmol, 1 equiv.) or $FeSO_4{\cdot}7H_2O$ (0.92 g, 3.3 mmol, 1 equiv.) in MeOH (10 mL). The solution was then stirred at 50 °C. After 30 min., the solvent was removed under reduced pressure, and the resulting crude solid was suspended in diethyl ether (200 mL). The suspension was subsequently filtered, and the collected solid was washed with additional diethyl ether (200 mL). Finally, the solid was collected to yield the title compound as a grayish-black color solid. Yield **2-Cl**: 85%; **2-SO_4**: 84%.

Complex 2-Cl: ^{1}H NMR (500 MHz, CD_3OD) δ 9.19 (bs, 3H), 8.73 (d, J = 4.9 Hz, 3H), 8.66 – 8.57 (m, 12H), 7.90 (d, J = 16.4 Hz, 6H), 7.75 – 7.62 (m, 30H). $^{13}C\{^1H\}$ NMR (126 MHz, CD_3OD): δ 161.0, 157.6, 155.2, 150.6, 146.8, 135.2, 132.5, 130.4, 123.1, 120.3. HRMS (ESI$^+$, *m/z*): calcd. for $[C_{72}H_{54}N_{12}Fe]^{2+}$ 571.1967; found 571.1946.

Complex 2-SO_4: ^{1}H NMR**:** (500 MHz, CD_3OD) δ 9.24 (bs, 3H), 8.73 (d, J = 4.9 Hz, 3H), 8.59 (dd, J = 9.8, 6.6 Hz, 12H), 7.90 (d, J = 11.1 Hz, 6H), 7.80 – 7.69 (m, 21H), 7.67 – 7.59 (m, 9H). $^{13}C\{^1H\}$ NMR (126 MHz, CD_3OD): δ 161.0, 157.6, 155.0, 150.6, 146.4, 135.1, 132.5, 130.4, 123.3, 120.3. HRMS (ESI$^+$, *m/z*): calcd. for $[C_{72}H_{54}N_{12}Fe]^{2+}$ 571.1967; found 571.1946.

Synthesis of complex 3

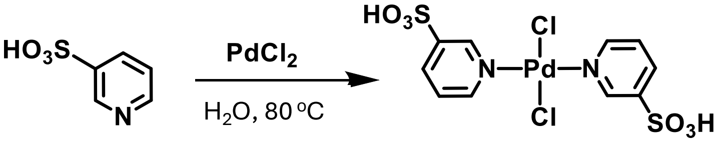

To an aqueous solution (10 mL) of pyridine-3-sulfonic acid (1.60 g, 10 mmol, 2 equiv.), an aqueous solution (5 mL) of palladium (II) chloride (0.88 g, 50 mmol, 1 equiv.) was added. The resulting reaction mixture was heated at 80 °C for 2 h and subsequently filtered to remove any insoluble particulates. Hereafter, the solvent was removed under reduced pressure to obtain the title compound as a brown solid. Yield: 80%. Single crystals suitable for Single X-ray diffraction (SC-XRD) could be obtained by slow evaporation of H_2O from a concentrated aqueous solution of **3**. ^{1}H NMR (300 MHz, D_2O) δ 9.13 (s, 2H), 8.88 (d, J = 5.6 Hz, 2H), 8.31 (d, J = 8.1 Hz, 2H), 7.65 (dd, J = 8.0, 5.8 Hz, 2H). ^{13}C{^{1}H} NMR: (126 MHz, D_2O) δ 154.6, 149.7, 141.2, 137.1, 126.2. MS (ESI^{+}), *m/z*: calcd. for $[C_{10}H_{10}ClN_2O_6S_2Pd]^{+}$ 460.18; found 460.87.

Single-crystal X-ray diffraction of complex 3. A single crystal suitable for X-ray diffraction was coated with Paratone oil (Hampton Research, CA, USA), mounted on a MiTeGen loop and flash frozen in liquid nitrogen. Diffraction data were recorded on Rigaku Synergy system equipped with a Dectris Pilatus 300K CdTe detector. Data were measured with MoKα radiation at 100K. The data were collected and processed with Rigaku OD CrysAlisPro 1.171.42.54a.[3] The structure was determined by direct methods using SHELXT-2018 and refined with SHELXL-2016/4 within Olex2.[4-6] The crystal data and the structural refinement are summarized in **Table S1** (CCDC 2362986, CIF V578).

Table S1. Single-crystal X-ray diffraction data of complex **3**.

CIF	V578
CCDC	2362986
Crystal description	Yellow plate
Source	Rigaku XtaLab Synergy
Empirical formula	$C_{10}H_8Cl_2N_2O_6PdS_2 + 8(H_2O)$
Formula weight (g/mol)	637.73
Temperature (K)	100
Wavelength (Å)	0.71073
Crystal system	triclinic
Space group	*P-1*
a (Å)	7.74914(9)
b (Å)	8.10458(12)
c (Å)	20.1773(2)
a°	91.5787(10)
b°	96.3016(10)
g°	112.3610(12)
Volume (Å^3)	1161.52(3)
Z	2
Density calculated (Mg/m^3)	1.824
Absorption coefficient (mm^{-1})	1.275
F(000)	644
Theta range for data collection (°)	2.037 to 27.483
Reflection collected (Unique)	53411(5322)
R_{int}	0.0415
Completeness %	99.9
Data/restraints/parameters	5322 /18/ 335
Goodness-of-fit on F^2	0.999
Final R [I>2s(I)]	R1 = 0.0284, wR2 = 0.0750
R (all data)	R1 = 0. 0298, wR2 = 0.0759
Largest diff. peak and hole (e$_\cdot$ Å^{-3})	1.955 and -1.676

Formation of Molecular Assemblies MA1 and MA2 by Spin Coating. Molecular assemblies were prepared by alternating spin coating of aqueous solutions of **3** (4.0 mM) and solutions of either **1-SO$_4$** (0.6 mM) or **2-SO$_4$** (0.6 mM). For **2-SO$_4$**, a H_2O/MeOH mixture (95:5 v/v) was used. Assembly formation was initiated by drop casting a solution of **3** (0.6-0.7 mL, 4.0 mM) onto the substrate, followed by spinning at 500 rpm for 10 s and then at 1000 rpm for 60 s. After a stationary period of 80 s, a solution of **1-SO$_4$** (0.6-0.7 mL, 0.6 mM) for **MA1** or **2-SO$_4$** (0.6-0.7 mL, 0.6 mM) for **MA2** was drop cast onto the substrate, which was then spun using the same protocol. Next, the substrates were immersed in methanol (25 mL) for 30 s and dried under a gentle stream of air. A single deposition cycle consists of one layer of **3** followed by one layer of **1-SO$_4$** or **2-SO$_4$**. For **MA1**, the number of deposition cycles was repeated 26 times, while for **MA2,** it was repeated 18 times in order to obtain the completed molecular assemblies.

Formation of Molecular Assemblies MA3 and MA4 by Spin Coating. The assemblies were obtained by alternatingly spin-coating aqueous solutions of Na_2PdCl_4 (4.0 mM) followed by aqueous solutions of complex **1-SO$_4$** (0.6 mM) or complex **2-SO$_4$** (0.6 mM). Note that for **2-SO$_4$** a mixture of H_2O:MeOH (95%:5% v/v) was used. Formation of the molecular assemblies was initiated by drop casting a solution of Na_2PdCl_4 (0.6-0.7 mL; 4.0 mM) onto the substrate, whereafter the substrate was spun at 500 rpm for 10 s, followed by 1000 rpm for 60 s. Hereafter, a solution (0.6-0.7 mL; 0.6 mM) of the **1-SO$_4$** (**MA3**) or **2-SO$_4$** (**MA4**) was drop casted after 80 s onto the substrates, which were again spun according to the above-mentioned protocol. Next, the substrates were immersed in methanol (0.6-0.7 mL) for 30 s and dried under a gentle stream of air. The deposition of Na_2PdCl_4 (**4**) followed by the deposition of **1-SO**$_4$ or **2-SO$_4$** is referred to as a single deposition cycle. For the formation of **MA3,** the deposition cycle was repeated 26 times, whereas for the formation of **MA4**, the deposition cycle was repeated 16 times, respectively.

Formation of Molecular Assemblies MA5 and MA6 by Spray Coating. The assemblies were obtained by automated and alternatingly ultrasonic spray coating of a methanol solution of **3** (1.0 mM) and of complex **1-SO$_4$** or **2-SO$_4$** in methanol (0.2 mM), respectively. The coatings were formed on FTO/glass (2 cm × 2 cm) at an atomization pressure of 1.03 or 1.30 kPa. The nozzle-to-substrate distance was 5.5 cm, and the nozzle was moved in a preprogrammed pattern along the *X* and *Y* directions at a speed of 5 mm/s and with a flow rate of 0.6 mL/min at room temperature

(~23 °C). The aqueous solution of **3** (1.0 mM) was sprayed onto the substrate (3 passes), which was followed by spraying (3 passes) the solutions complex **1-SO$_4$** or **2-SO$_4$** in methanol (0.2 mM). This deposition sequence was repeated six times to generate **MA5** and **MA6**. The substrates after formation of the **MAs** were removed from the spray coater and immersed in methanol for 30 s and dried under a gentle stream of air.

Fabrication of Electrochromic Devices. The FTO/glass substrates coated with **MA1-MA4** served as the working electrode, and a bare substrate (FTO/glass) served as both the reference and counter electrodes. A frame of 210-μm-thick double-sided tape (3M 9088) was attached to the working electrode (2 cm × 2 cm) leaving an exposed edge (1-2 mm) for silver paste or copper tape contacts. Contacts were also connected to an edge (1-2 mm) of the counter electrode. The electrodes were placed with the two conducting faces facing each other. The electrolyte gel (90:7:3 wt% ACN/PMMA/lithium perchlorate salt) was injected using a syringe between the two electrodes.

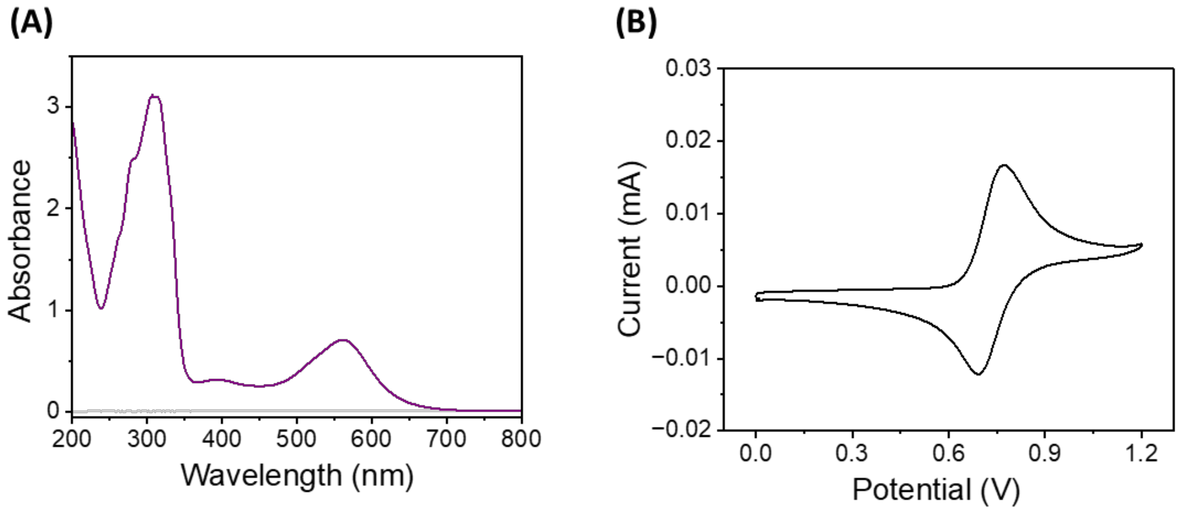

Figure S1. (A) UV-Vis spectrum of complex **1-Cl** (0.03 mM) in water. (B) Cyclic voltammogram of complex **1-Cl** (0.125 mM|) in water, recorded at a scan rate of 0.1 V/s, with NH_4Cl (0.1 M) as the supporting electrolyte.

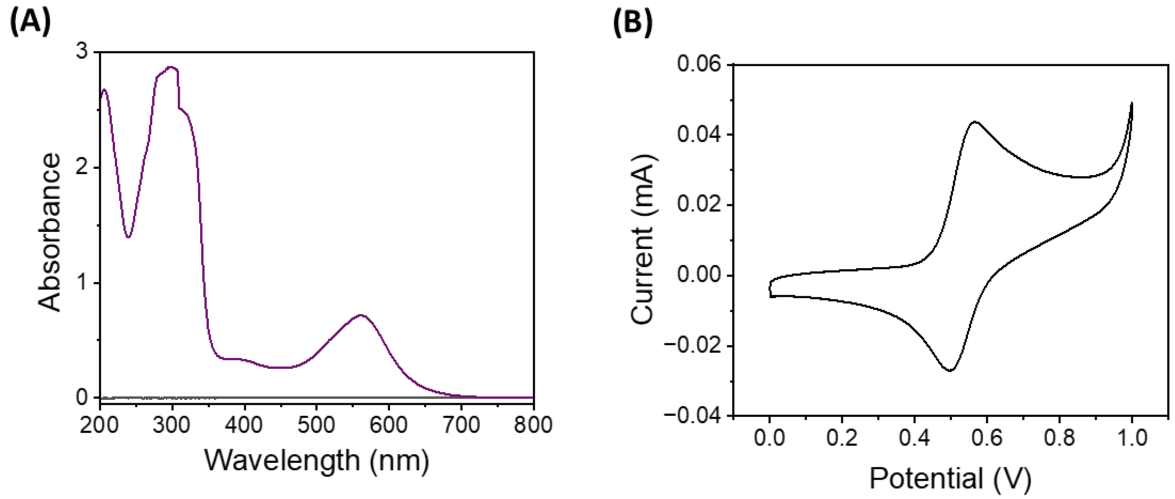

Figure S2. (A) UV-Vis spectrum of complex **1-SO$_4$** (0.03 mM) in water. (B) Cyclic voltammogram of complex **1-SO$_4$ (**0.125 mM) in water, recorded at a scan rate of 0.1 V/s, with $(NH_4)_2SO_4$ (0.1 M) as the supporting electrolyte.

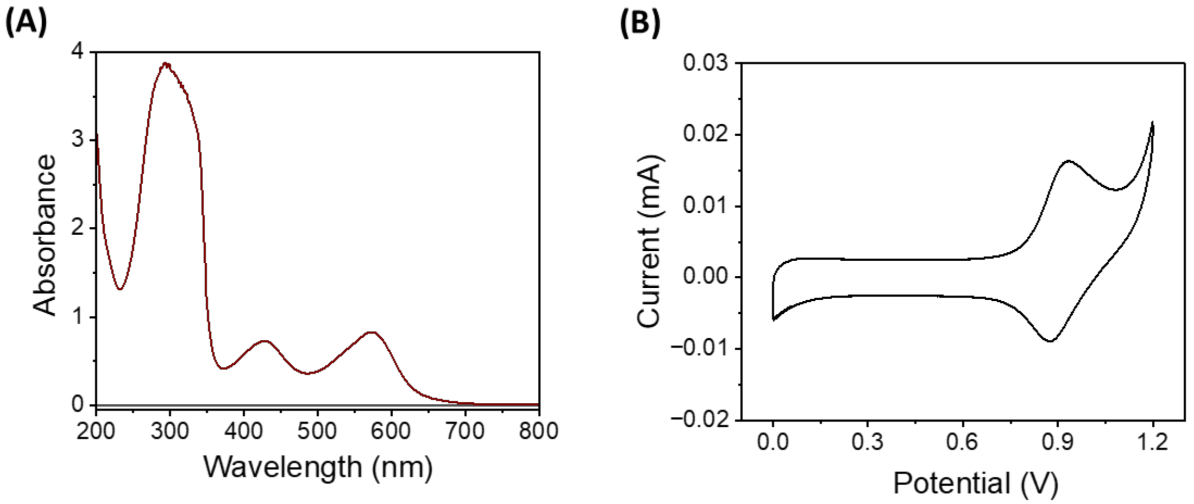

Figure S3. (A) UV-Vis spectrum of complex **2-Cl** (0.03 mM) in water. (B) Cyclic voltammogram of complex **2-Cl** (0.125 mM) in water, recorded at a scan rate of 0.1 V/s, with NH_4Cl (0.1 M) as the supporting electrolyte.

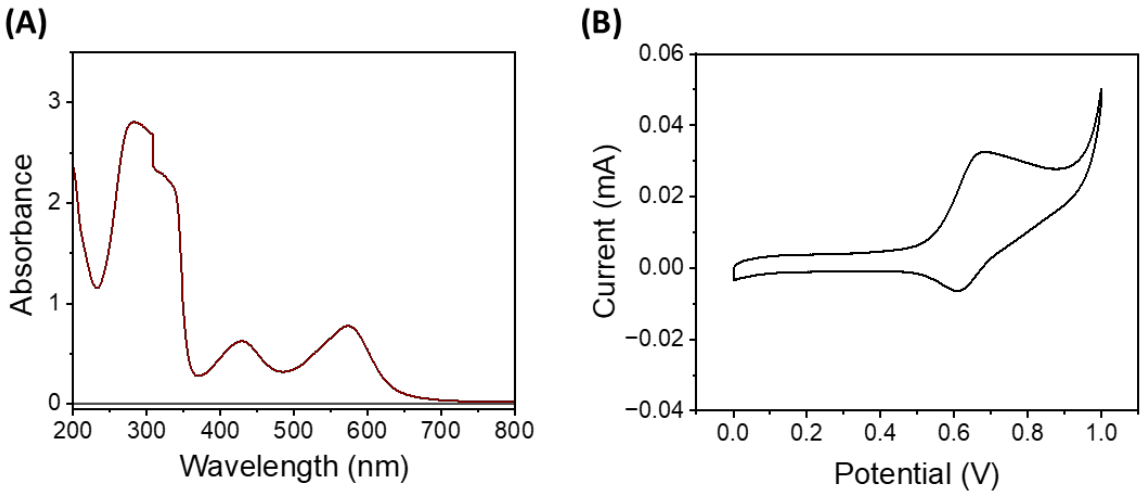

Figure S4. (A) UV-Vis spectrum of complex **2-SO$_4$** (0.03 mM) in water. (B) Cyclic voltammogram of complex **2-SO$_4$** (0.125 mM) in water, recorded at a scan rate of 0.1V/s with $(NH_4)_2SO_4$ (0.1 M) as the supporting electrolyte.

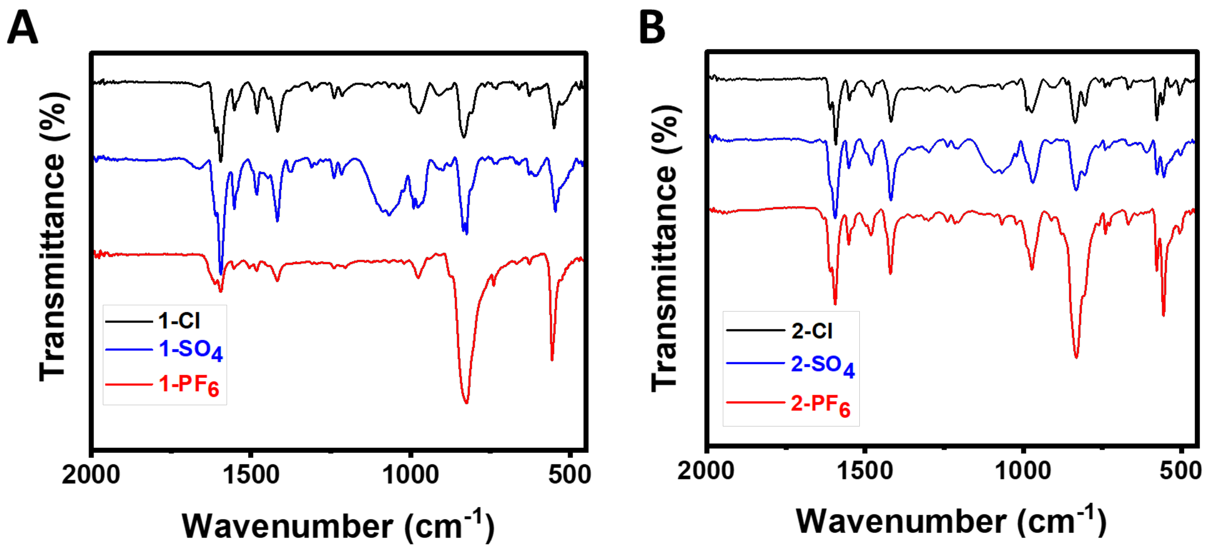

Figure S5. Fourier-transform infrared spectroscopy (FTIR) of (A) complex **1** and (B) complex **2**, with different counter ions.

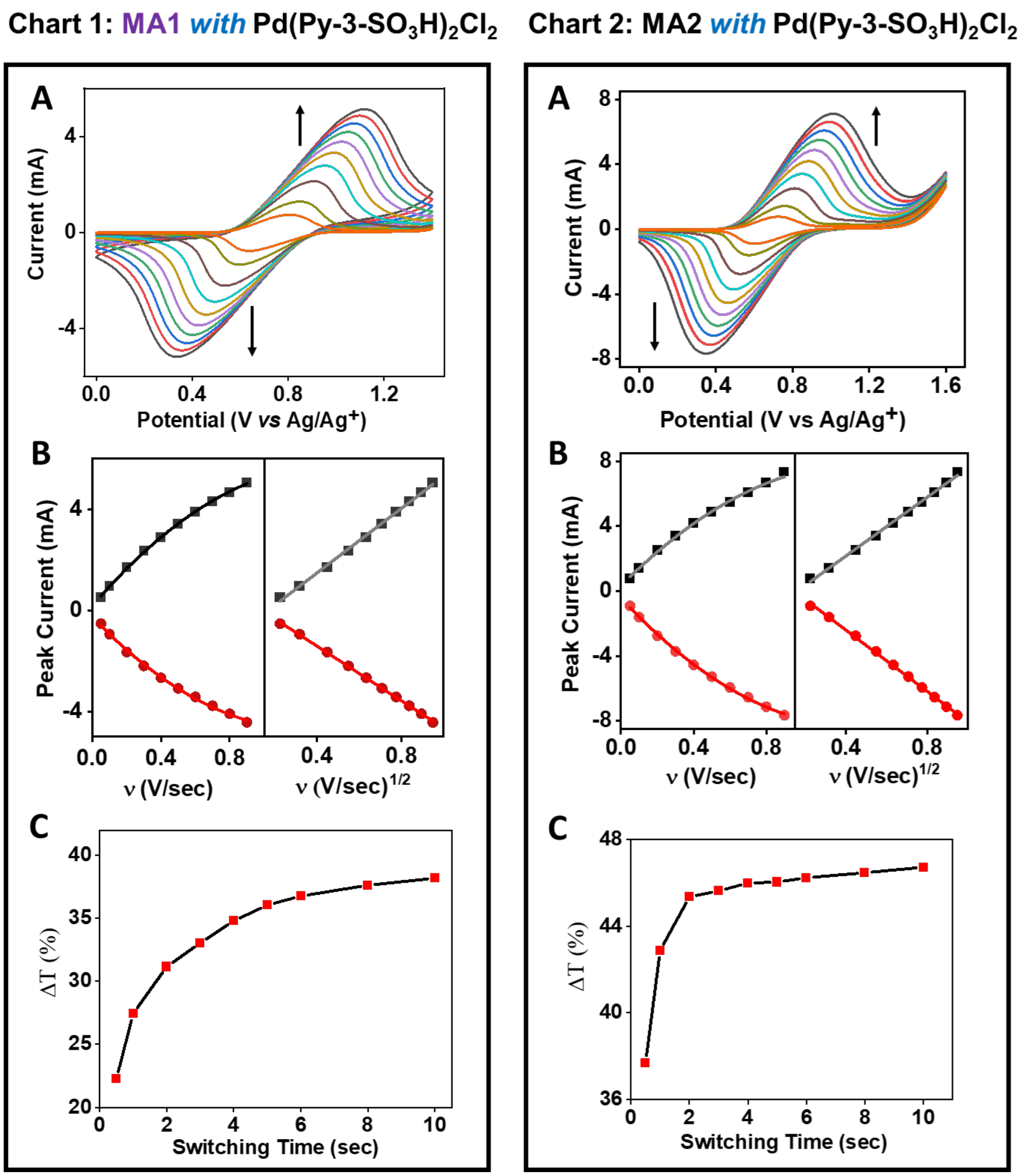

Figure S6. **Chart 1** [**MA1**|FTO/glass] and **Chart 2** [**MA2**|FTO/glass]. (A) Cyclic voltammograms (CVs) with scan rates (ν) of 0.05-0.9 V/s. (B) Exponential (left) and linear (right) correlations between the peak currents (I) and ν or $\nu^{1/2}$, during oxidation (black) and reduction (red) ($R^2 > 0.99$ for all fits). (C) Contrast ratio (ΔT) vs switching time. $LiClO_4$ (0.1 M) in H_2O was used as electrolyte solution. Pt and Ag wires were used as counter and reference electrodes, respectively.

Chart 1. MA3 *with* Na_2PdCl_4 linker

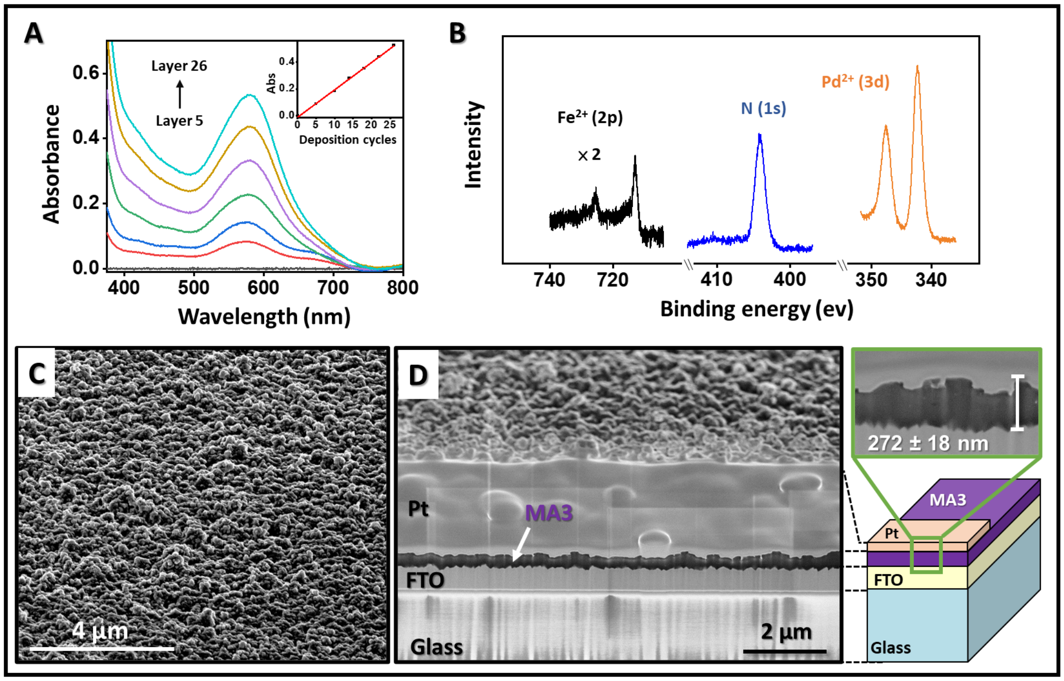

Chart 2. MA4 *with* Na_2PdCl_4 linker

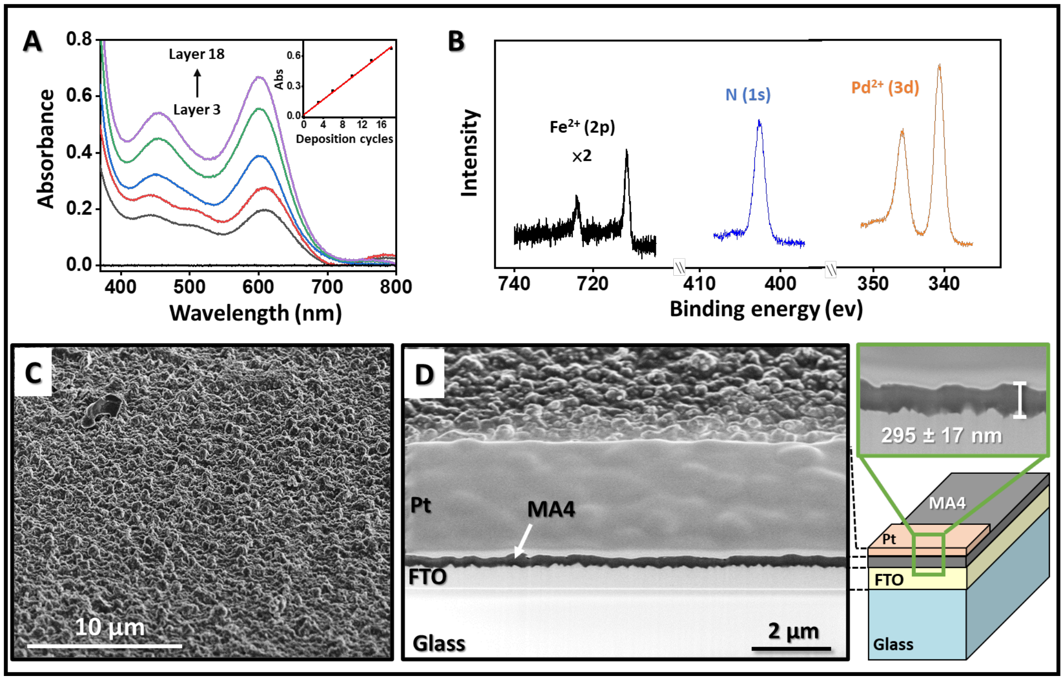

Figure S7. **Chart 1** [**MA3**|FTO/glass] and **Chart 2** [**MA4**|FTO/glass]. (A) Absorption spectra after several deposition cycles. Bare FTO was used for the baseline (black). Inset: Absorbance intensity of the MLCT bands (**MA3**: λ_{max} = 579 nm) (**MA4**: λ_{max} = 596 nm) vs the number of deposition cycles. (B) X-ray photoelectron spectroscopy (XPS) spectra. (C) Scanning electron microscopy (SEM) images of the surfaces. (D) Cross sections generated by milling with a 30 keV Ga^+ focused ion beam (FIB). The surface was first covered with a 3-nm thick layer of iridium followed by a Pt coating to prevent ion beam damage.

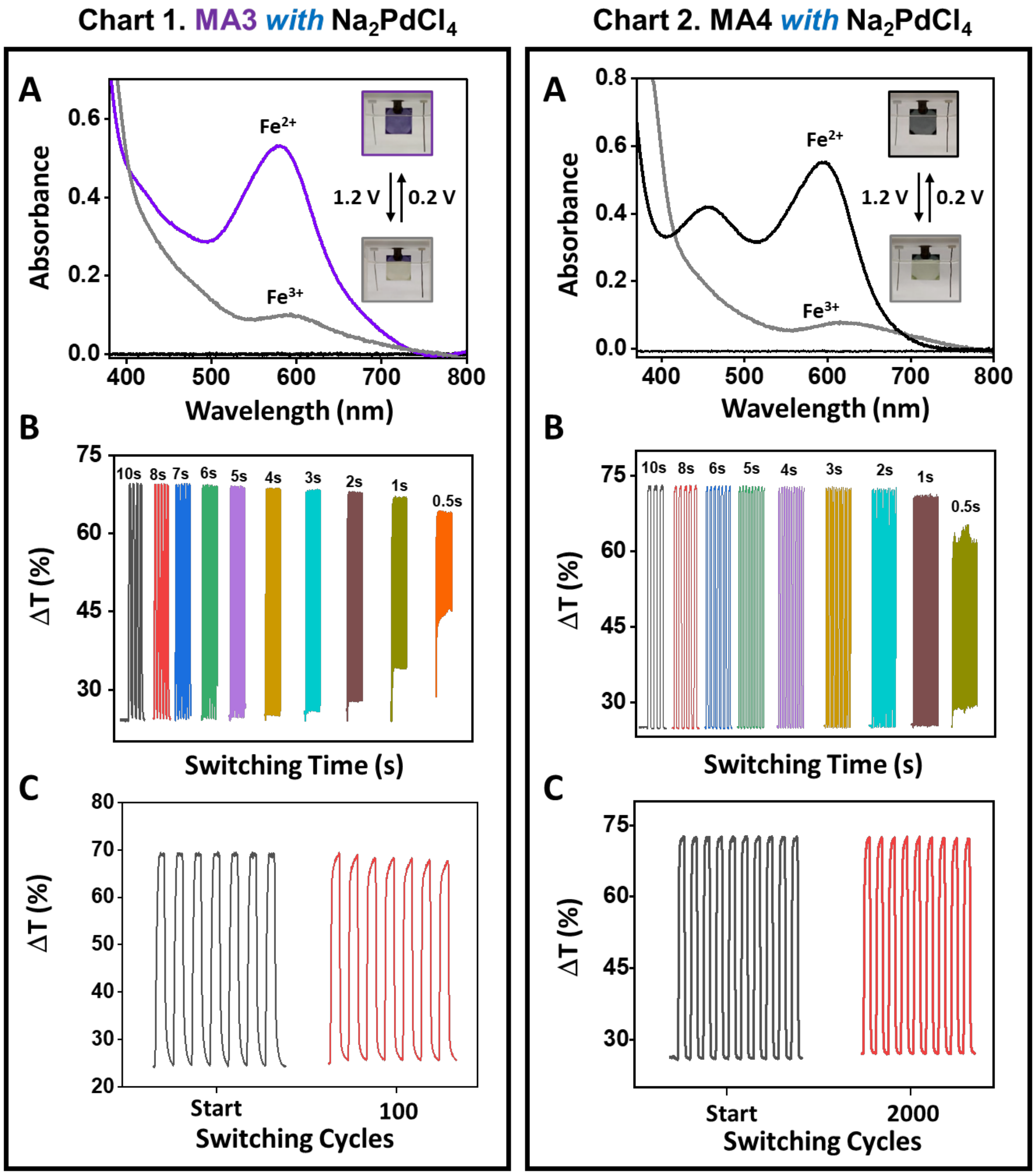

Figure S8. Electrochromic performance of [**MA3**|FTO/glass] and [**MA4**|FTO/glass] in 0.1 M $LiClO_4/H_2O$ electrolyte solution using Pt wire and Ag wire, as counter and reference electrodes, respectively. The **MA3** and **MA4** were prepared by alternating spin coating of aqueous solutions of Na_2PdCl_4 (**4**) and complex **1-SO$_4$** and **2-SO$_4$**, respectively. **Chart 1:** [**MA3**|FTO/glass] and **Chart 2:** [**MA4**|FTO/glass]: (A) Absorption spectra showing the reduced (0.2 V) and oxidized (1.2 V) states. FTO/glass was used for the baseline (black). (Inset) Photographs of the colored (Fe^{2+}, 0.2 V) and bleached (Fe^{3+}, 1.2 V) states. (B) Spectroelectrochemical (SEC) measurements at different switching times. (C) SEC stability measurements. For (B) and (C) Double potential steps: 0.2 V to 1.2 V, λ_{max} = 579 nm (**Chart 1**) and λ_{max} = 596 nm (**Chart 2**).

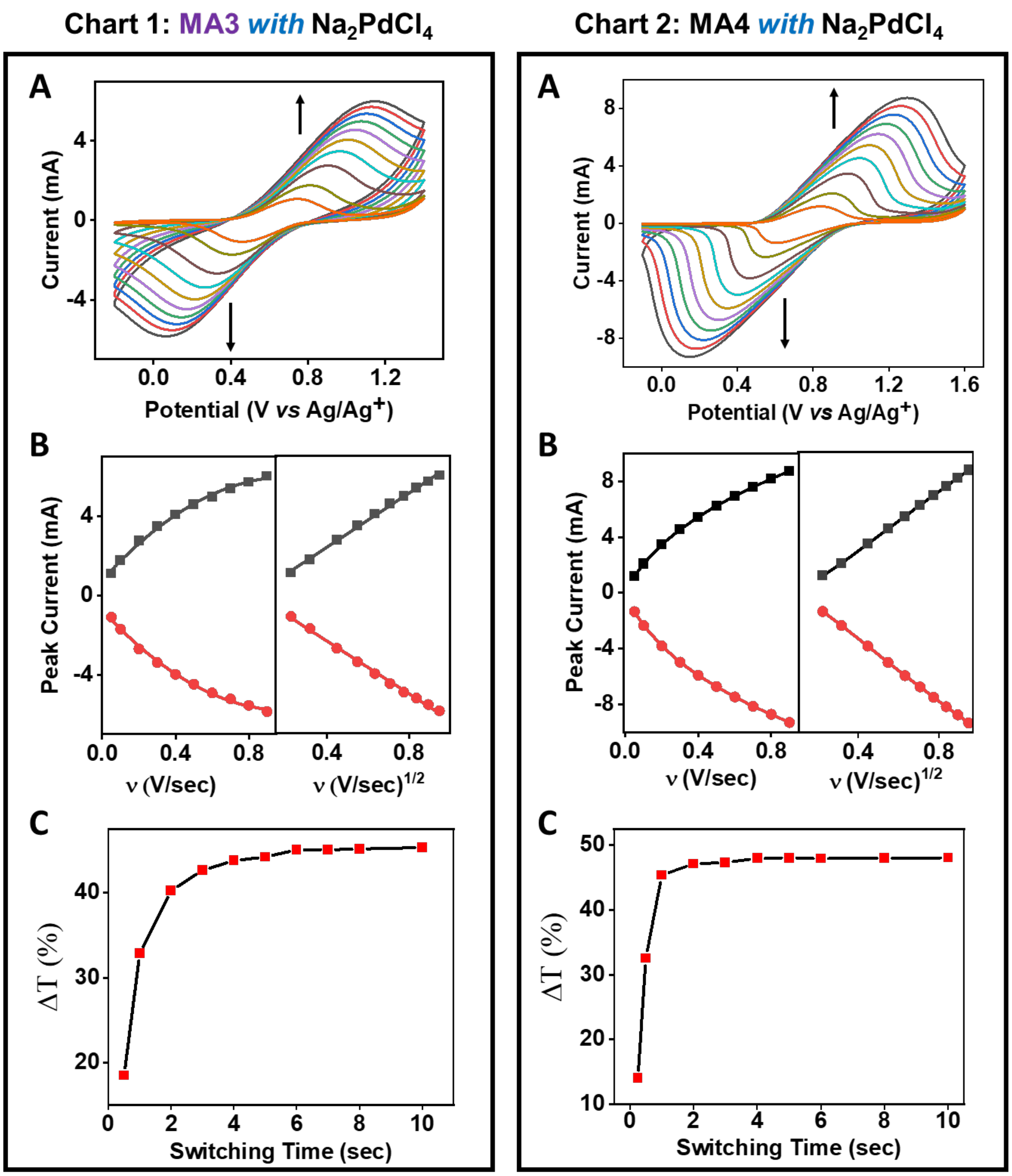

Figure S9. Spectroelectrochemical (SEC) performance of [**MA3**|FTO/glass] and [**MA4**|FTO/glass] in 0.1 M $LiClO_4/H_2O$ electrolyte solution using Pt wire and Ag wire, as counter and reference electrodes, respectively. The **MA3** and **MA4** were prepared by alternating spin coating of aqueous solutions of Na_2PdCl_4 (**4**) and complex **1-SO₄** and **2-SO₄**, respectively. **Chart 1:** [**MA3**|FTO/glass] and **Chart 2:** [**MA4**|FTO/glass]: (A) Cyclic voltammograms (CVs) with scan rates of 0.05-0.9 V/s. (B) Exponential and linear correlations between the peak currents (I) and scan rates (ν) (left), and I and $\nu^{1/2}$ (right) respectively, during oxidation (grey) and reduction (red) (R^2 > 0.99 for all fits). (C) Dependence of the contrast ratio (ΔT) on the switching time.

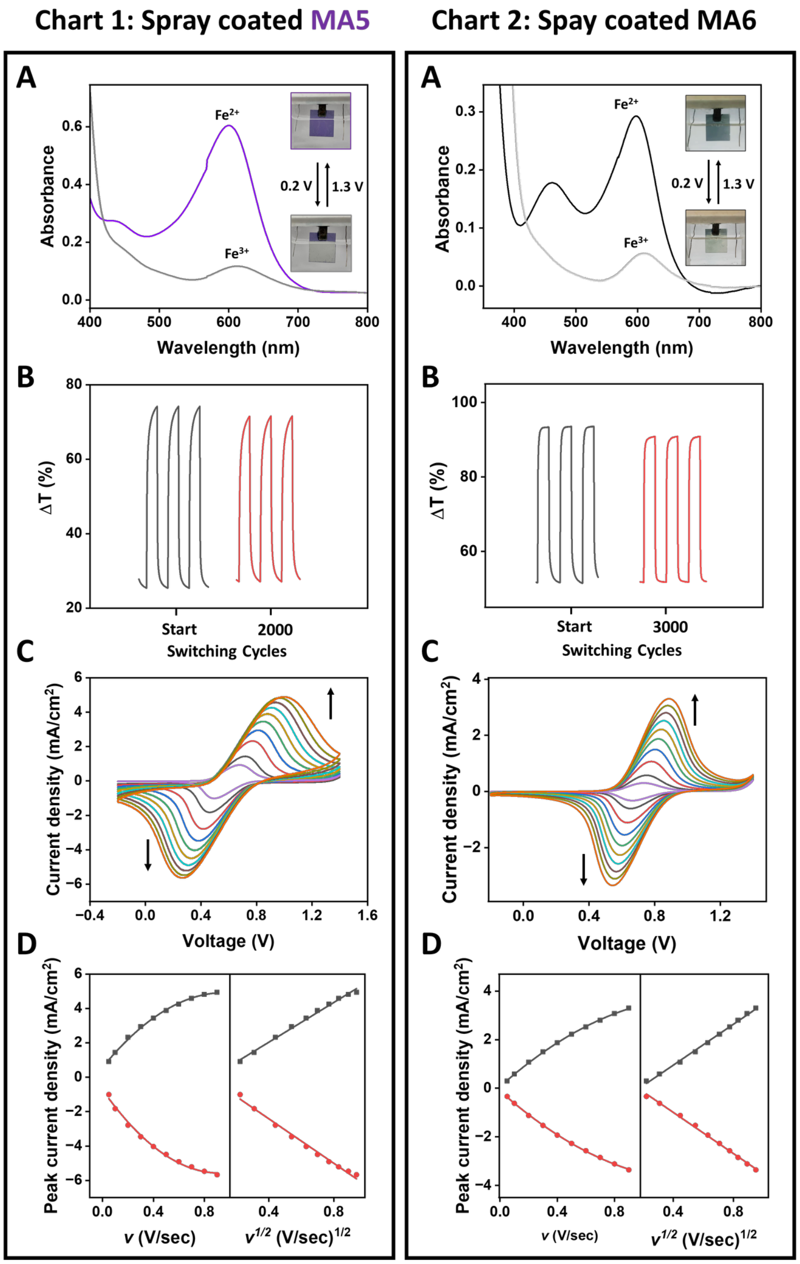

Figure S10. Spectroelectrochemical (SEC) performance of [**MA5**|FTO/glass] and [**MA6**|FTO/glass] in 0.1 M $LiClO_4/H_2O$ electrolyte solution using Pt wire and Ag wire, as counter and reference electrodes, respectively. **MA5** and **MA6** were prepared by alternating ultrasonic spray coating of methanol solutions of *trans*-[Pd(3-SO_3H-py)$_2$$Cl_2$] and complex **1-SO$_4$** and **2-SO$_4$**, respectively. **Chart 1:** [**MA5**|FTO/glass] and **Chart 2:** [**MA6**|FTO/glass]: (A) Absorption spectra showing the reduced (0.2 V) and oxidized (1.3 V) states. FTO/glass was used for the baseline (black). (Inset) Photographs of the colored (Fe^{2+}, 0.2 V) and bleached (Fe^{3+}, 1.3 V) states. (B) SEC stability measurements at λ_{max} = 579 nm (**Chart 1**) and λ_{max} = 596 nm (**Chart 2**) using double potential steps: 0.2 V to 1.3 V. (C) Cyclic voltammograms (CVs) with scan rates of 0.05-0.9 V/s. (D) Exponential and linear correlations between the peak currents (I) and scan rates (v) (left), and I and $v^{1/2}$ (right) respectively, during oxidation (grey) and reduction (red) (R^2 > 0.99 for all fits).

Table S2. Comparison of various parameters for the molecular assemblies **MA1-MA4**.

Parameters	Cross linker (**3**): *trans*-[Pd(3-SO_3H-py)$_2Cl_2$]		Cross linker (**4**): Na_2PdCl_4	
	**MA1**	**MA2**	**MA3**	**MA4**
Color	Purple	Gray	Purple	Gray
λ_{max} (nm)	579	598	579	598
ΔT%	39	46	46	47
Stability (95%) (in solution)	1800	2000	100	2000
$E_{1/2}$ (V)	0.720	0.668	0.607	0.723
Diffusion Coefficient (cm^2/s)	Ox: 1.5×10^{-8}	Ox: 2.4×10^{-9}	Ox: 2.1×10^{-8}	Ox: 1.8×10^{-9}
	Red: 1.6×10^{-8}	Red: 2.0×10^{-9}	Red: 1.9×10^{-8}	Red: 1.2×10^{-9}
Switching time for Oxidation (s)	0.9	1.0	1.4	1.8
Switching time for Reduction (s)	0.8	0.9	1.3	1.5
molecules/cm^2 (CV)	0.7×10^{16}	0.9×10^{16}	1.0×10^{16}	1.2×10^{16}
molecules/cm^2 (UV-Vis)	0.9×10^{16}	1.0×10^{16}	1.8×10^{16}	1.9×10^{16}

References

1. Malik, N.; Elool Dov, N.; de Ruiter, G.; Lahav, M.; van der Boom, M. E. On-surface self-assembly of stimuli-responsive metallo-organic films: Automated ultrasonic spray-coating and electrochromic devices. *ACS Appl. Mater. Interfaces* **2019**, *11*, 22858–22868.
2. Shankar, S.; Lahav, M.; van der Boom, M. E. Coordination-based molecular assemblies as electrochromic materials: ultra-high switching stability and coloration efficiencies. *J. Am. Chem. Soc.* **2015**, *137*, 4050–4053.
3. Rigaku Oxford Diffraction. CrysAlisPro Software System, version 1.171.42.54a; Rigaku Oxford Diffraction: Oxford, U.K., **2022**.
4. Sheldrick, G. M. SHELXL-2018: Program for the Solution of Crystal Structures; University of Göttingen: Göttingen, Germany, **2018**.
5. Sheldrick, G. M. SHELXT – Integrated Space-Group and Crystal-Structure Determination. *Acta Crystallogr. Sect. A* **2015**, *71*, 3–8.
6. Dolomanov, O. V.; Bourhis, L. J.; Gildea, R. J.; Howard, J. A. K.; Puschmann, H. OLEX2: A Complete Structure Solution, Refinement and Analysis Program. *J. Appl. Crystallogr.* **2009**, *42*, 339–341.
